# Elevated apoptosis impairs epithelial cell turnover and shortens villi in TNF-driven intestinal inflammation

**DOI:** 10.1038/s41419-018-1275-5

**Published:** 2019-02-06

**Authors:** Aimée Parker, Laura Vaux, Angela M. Patterson, Amisha Modasia, Daniele Muraro, Alexander G. Fletcher, Helen M. Byrne, Philip K. Maini, Alastair J. M. Watson, Carmen Pin

**Affiliations:** 1 Gut Health and Food Safety Research Programme, Quadram Institute Bioscience, Norwich, United Kingdom; 2 Wellcome Sanger Institute, Hinxton, United Kingdom; 3 School of Mathematics and Statistics, University of Sheffield, Sheffield, United Kingdom; 4 Bateson Centre, University of Sheffield, Sheffield, United Kingdom; 5 Wolfson Centre for Mathematical Biology, Mathematical Institute, University of Oxford, Oxford, United Kingdom; 6 Norwich Medical School, University of East Anglia, Norwich, United Kingdom; 7 Drug Safety and Metabolism, IMED Biotech Unit, AstraZeneca, Cambridge, United Kingdom

## Abstract

The intestinal epithelial monolayer, at the boundary between microbes and the host immune system, plays an important role in the development of inflammatory bowel disease (IBD), particularly as a target and producer of pro-inflammatory TNF. Chronic overexpression of TNF leads to IBD-like pathology over time, but the mechanisms driving early pathogenesis events are not clear. We studied the epithelial response to inflammation by combining mathematical models with in vivo experimental models resembling acute and chronic TNF-mediated injury. We found significant villus atrophy with increased epithelial cell death along the crypt-villus axis, most dramatically at the villus tips, in both acute and chronic inflammation. In the acute model, we observed overexpression of TNF receptor I in the villus tip rapidly after TNF injection and concurrent with elevated levels of intracellular TNF and rapid shedding at the tip. In the chronic model, sustained villus atrophy was accompanied by a reduction in absolute epithelial cell turnover. Mathematical modelling demonstrated that increased cell apoptosis on the villus body explains the reduction in epithelial cell turnover along the crypt-villus axis observed in chronic inflammation. Cell destruction in the villus was not accompanied by changes in proliferative cell number or division rate within the crypt. Epithelial morphology and immunological changes in the chronic setting suggest a repair response to cell damage although the villus length is not recovered. A better understanding of how this state is further destabilised and results in clinical pathology resembling IBD will help identify suitable pathways for therapeutic intervention.

## Introduction

Inflammatory bowel disease (IBD) is associated with excessive epithelial death in the ileum and colon^1^. Recent findings suggest a primary role for focal injury of the epithelial lining and selection for aggressive microbial communities preceding the establishment of Crohn’s-like ileitis^2–4^. Likewise, the murine dextran sodium sulfate (DSS) colitis model highlights the importance of the severity of epithelial injury in the establishment of IBD. Depending on the DSS dose, animals present either severe intestinal injury with impaired “mucosal healing” and fatality, or mild injury with rapid restoration of mucosal integrity^5,6^. Ultimately, re-establishment of the epithelial barrier leads to sustained clinical remission and resection-free survival in IBD patients^7^.

TNF is a cytokine produced by immune, mesenchymal and epithelial cells, and regulates the epithelial barrier in multiple ways, including mucus secretion, barrier permeability, proliferation/differentiation and wound healing^8–10^. A single exogenous high dose of TNF induces transient intestinal damage with rapid epithelial cell apoptosis, predominantly at villus tips, villus shortening, fluid exudation into the gut lumen, and diarrhoea^8,11–13^. Animal models with persistent elevated TNF exhibit IBD-like inflammatory changes in the mucosa and are widely used to study intestinal chronic inflammatory processes^3,14,15^. Such models reveal the role of epithelial cells as targets and producers of TNF in apoptotic death, leading to barrier breach and ultimately to IBD-like pathology^16–18^.

Numerous studies using TNFRI and TNFRII knockout mouse models suggest TNF-induced cell apoptosis in the small intestine is triggered primarily through TNFRI signalling^11,13,18–21^. although heterogeneous responses are detected upon differences in signal transduction downstream of the receptor binding^22–24^. TNFRII can play an additive role in enterocyte death^11,13^ or diverse roles in epithelial cell survival, proliferation and migration, and immune regulation^25–28^.

**Fig. 1:**
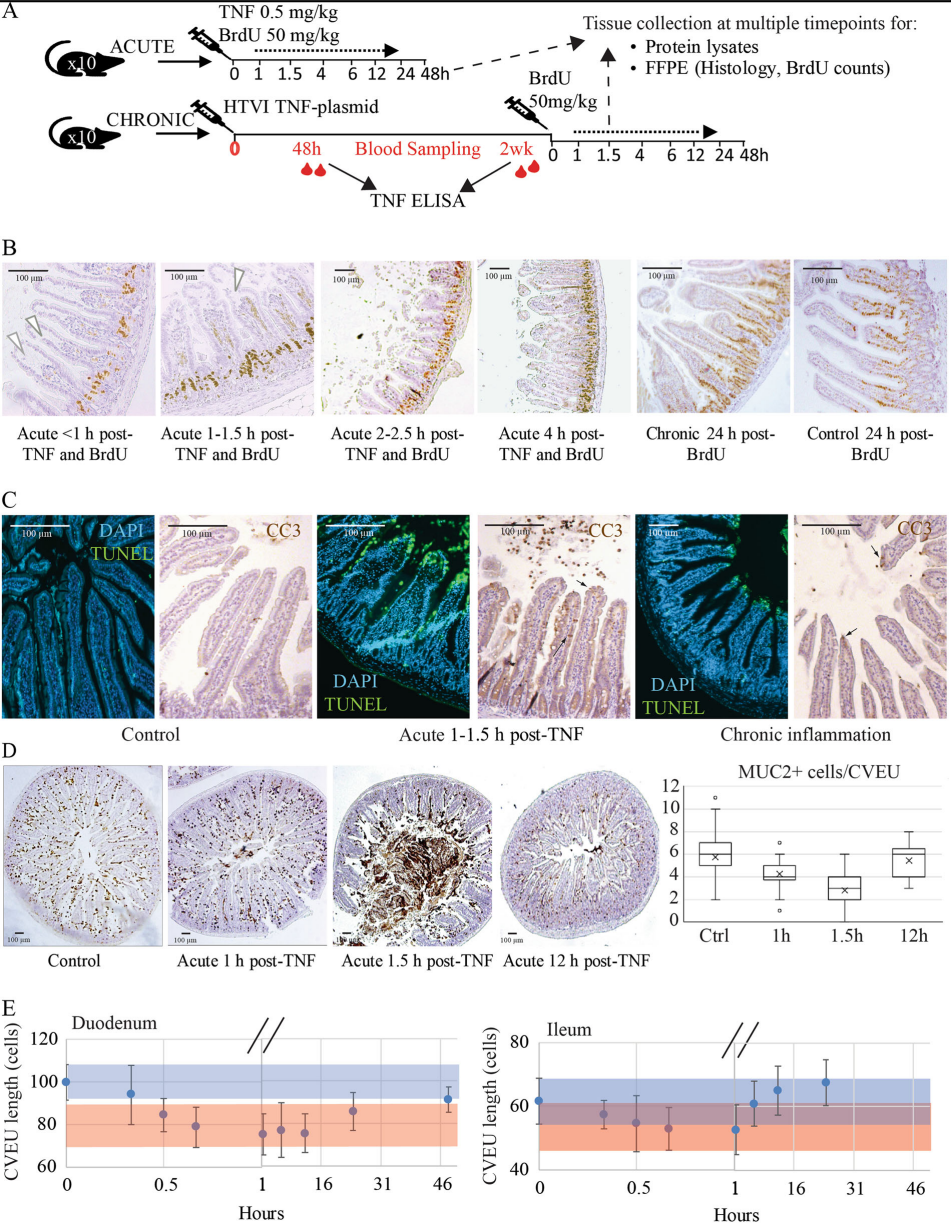
Changes in the small intestinal epithelium of acute and chronic TNF-mediated injury mouse models. **a** Schematic of experimental treatment and sampling timeline for acute and chronic TNF-mediated inflammatory injury. **b** Morphology of duodenal sections illustrating epithelial disruption 1–4 h following a high-dose pulse of TNF (acute model) with concomitant BrdU administration (brown staining), counterstained with Haematoxylin (blue/purple). Arrows indicate the hollow villus tips following stromal retraction induced by TNF and the constriction of the epithelium over the stroma preceding the shedding of the tip, which is re-epithelised at 4 h post-TNF. The epithelium in healthy and chronic inflammation models exhibits standard morphological appearance. Progression of BrdU-labelled cells on the CVEU over time was used to quantify cell dynamics in later analyses. **c** Images of TUNEL and cleaved-Caspase-3 (CC3) labelled duodenum sections illustrating labelling similarity and differences in cell death intensity along the CVEU of healthy and inflammation mouse models. **d** Representative images and quantification of cells staining positive for goblet cell mucin (MUC2) in small intestine of control and acute inflammation mouse model at 1, 1.5, and 12 h post-TNF delivery. **e** Plot symbols show the decrease and recovery of the CVEU length (average number of cells ± standard deviation) in duodenum and ileum over time following the administration of one high-dose pulse of TNF (acute inflammation). Continuous blue and red bands show the average ± standard deviation of the CVEU length in control conditions and the chronic inflammation model, respectively

We here investigated epithelial cell dynamics in the small intestine of experimental mouse models of acute and chronic intestinal inflammation. Acute inflammation was induced by a single intraperitoneal delivery of recombinant TNF, while chronic inflammation was induced by delivery of a TNF-expressing plasmid, resulting in lower, but persistent, levels of circulating TNF (Fig. 1a). We studied two TNF-responsive regions^11,13,29^: the duodenum which, is usually not compromised by IBD, and the ileum, which exhibits typical lesions during IBD episodes. We combined cell labelling and tracking techniques with mathematical modelling to quantify cell dynamics along the crypt-villus epithelial unit (CVEU), a one-dimensional column of cells running from the base of a crypt to the tip of an adjoining villus^30,31^. We used Bromodeoxyuridine (BrdU) to quantify the progression of labelled cells along the CVEU, from which we inferred the absolute cell production rate, henceforth referred to as epithelial turnover. This rate quantifies the cell yield resulting from proliferation and death along the CVEU and differs from the number of cells generated per proliferative cell per unit time, which we referred to as division rate. Epithelial turnover depends on the number of proliferative cells, the division rate, and the rate at which cells die along the crypt-villus axis. To study each of these parameters in our experimental models, we combined mathematical models with BrdU S-phase cell labelling, Vincristine mitosis arrest, and TUNEL staining. Concurrently, we measured the intracellular concentration of TNF and the spatial distribution of TNF receptors along the CVEU. Applying this methodology, we aimed to gain insight into the loss of epithelial homoeostasis preceding IBD development.

## Results

### TNF causes villus atrophy and prolonged exposure results in IBD-associated immune changes

Following a single intraperitoneal delivery of rTNF (0.5 mg/kg) (Fig. 1a), intense cell death and shedding was seen at villus tips (Fig. 1b). This response peaked around 1–1.5 h post TNF administration, when the concentration of circulating TNF reached levels of 1200 pg/ml plasma (Fig. 1b). Shedding cells were TUNEL and cleaved-Caspase-3 (CC3) positive (Fig. 1c) indicating cell death by apoptosis. Goblet cell mucus depletion (MUC2 staining) was also evident at 1–1.5 h post induction (Fig. 1d). Tip cell shedding was preceded by retraction of the villus core and constriction of the villus tip epithelium (Fig. 1b). Within 2–4 h post-injection, the villus recovered its gross morphology, although not its original dimensions, while MUC2 depletion persisted after 12 h (Fig. 1d). Acute single delivery of doses at or below 0.25 mg/kg had no apparent effect on apoptotic shedding (data not shown).

We used a TNF-expressing plasmid, delivered by hydrodynamic tail vein injection (HTVI) to induce low-level TNF production in our chronic model of inflammation. We quantified circulating TNF at 48 h, 1 week and 2 weeks post-plasmid delivery and observed constant average values of 216 ± 82 pg/ml plasma, similar to those reported in genetically altered mouse models of TNF-driven intestinal damage, 90–430 pg/ml^4,14,15^. These relatively low TNF levels result in no effect if delivered as a single dose, but lead to pathology if maintained for longer periods^4,14,15^. Core retraction, tip constriction and shedding were not observed in the samples collected post-induction of chronic injury (Fig. 1b). However, villus length was significantly reduced to a similar extent as in the acute model (Fig. 1e). Chronic TNF-expressing mice exhibited decreased CVEU length for the duration of the treatment, while in the acute model the initial drastic reduction of villus length was followed by full recovery (Fig. 1e). Thus, although the villus tips were confluently covered by epithelial cells and the apoptotic index returned to control levels, the recovery of the villus tip was prevented in the chronic setting.

We studied the intestinal immune response by analysing expression of inflammation-associated proteins and assessing the mucosal immune cell composition with flow cytometry. Levels of CXCL5, MCP-1 and M-CSF, which are all involved in recruitment of immune cells to sites of inflammation, were increased in chronic inflammation (Fig. S1A). Flow cytometry analysis showed a shift from T-effector to T-regulatory profile in chronic inflammation, compared to acute TNF-injured and healthy mucosa (Figs S1B-S1C).

### Chronic inflammation decreases epithelial cell turnover

**Fig. 2:**
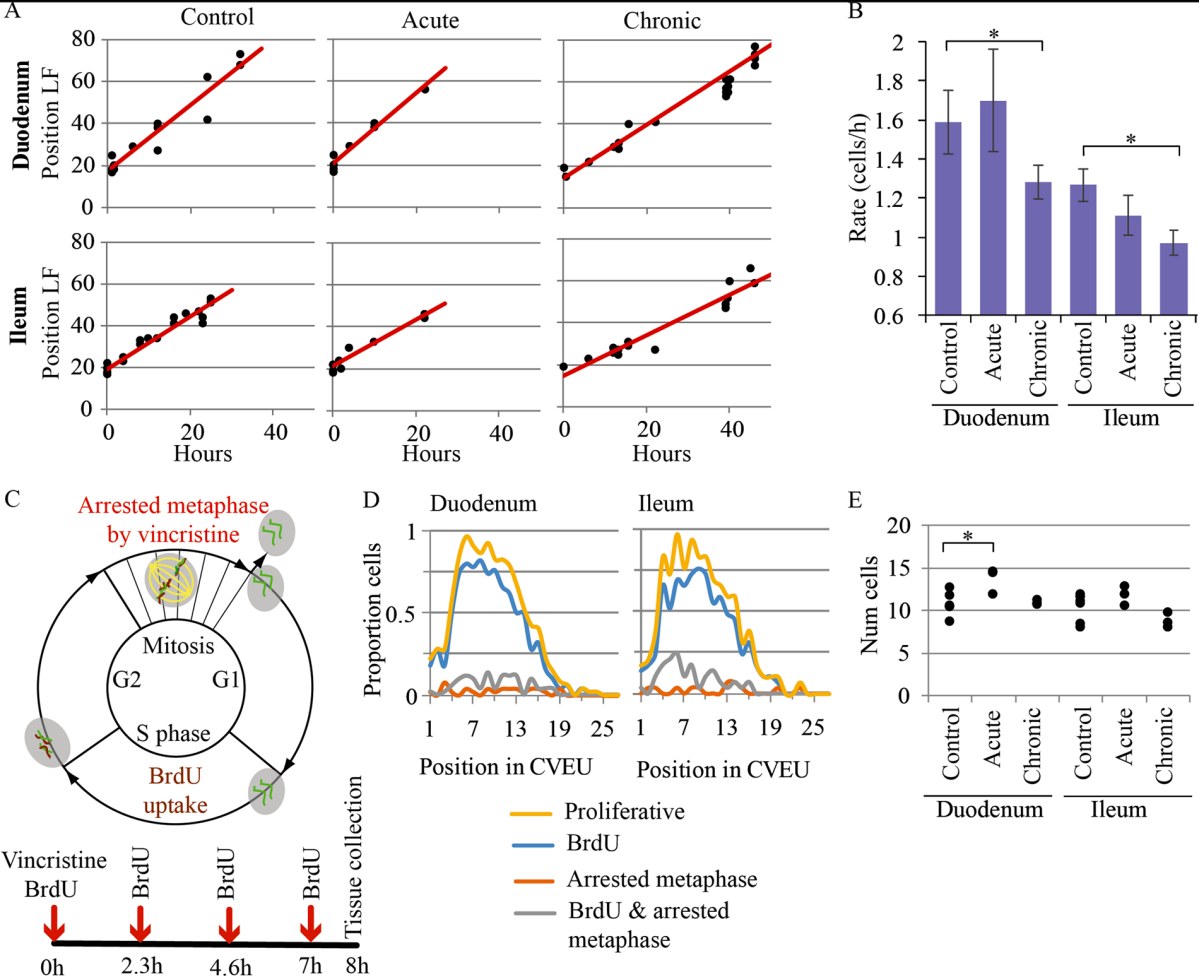
Quantification of the impact of chronic and acute inflammation on the CVEU cell production rate and on the size of the proliferative compartment. **a** Observed (symbols) and predicted (continuous lines; equation [1] in supplementary material) position of the BrdU-labelled front (LF) over time on the CVEU in homoeostasis and acute and chronic inflammation mouse models. **b** Comparison of the CVEU cell production rate (cells/h) in control conditions with that observed in chronic and acute inflammation conditions. Stars denote statistically significant differences. **c** Cartoon representative of the cell cycle with BrdU uptake and metaphase arrest by vincristine administration and administration schedule of vincristine and BrdU devised to detect proliferative cells. **d** Number of BrdU-labelled cells, cells in arrested metaphase, BrdU-labelled cells in arrested metaphase and proliferative cells at each position of the CVEU in duodenum and ileum of healthy mice. **e** Number of proliferative cells in the CVEU of duodenum and ileum in healthy and inflammation mouse models. Stars denote statistically significant differences between groups according to a Tukey test

To study whether inflammatory cell loss and villus shrinkage was accompanied by changes in cell production, we modelled the epithelium as a system of contiguous, non-compressible cells with an open boundary at the villus tip. This dictates that the excess of cells proliferating within the crypt migrate towards the tip, along the crypt-villus axis. Thus, after a single BrdU injection, the spatial progression of the labelled front along the CVEU reflects the absolute number of cells generated within the crypt per unit time, as previously reported^30,31^. We generated a BrdU labelling dataset by injecting a single dose of BrdU and sampling repeatedly in the following 48 h for both the acute and chronic models (Fig. 1a) and used it to fit equation (2) and estimate the epithelial turnover as described in the supplementary material. Figure 2a shows the predicted and observed progression of the BrdU-labelled cell front on the villus in each mouse model. Fits, diagnosis plots and posterior parameter estimates are shown in Fig. S2 and Table S1.

Although epithelial turnover was apparently unaffected by acute inflammation in either duodenum or ileum (Fig. 2b; Table S1), we consider this result inconclusive because the BrdU tracking period required for this estimation exceeded the duration of the acute inflammation process. On the other hand, cell production rates in chronic inflammation were significantly reduced compared to healthy intestine (Fig. 2b; Table S1), suggesting sustained inflammation inhibits the intestine’s ability to maintain healthy epithelial turnover. To validate our chronic setting, we used an equivalent model of long-term low-dose TNF delivered by subcutaneous osmotic minipumps, achieving similar plasma levels of 146 ± 97 pg/ml. We observed similar reduction in cell production in these two chronic models (Fig. S3A), supporting the hypothesis that relatively low levels of circulating TNF maintained for long periods results in decreased epithelial turnover.

As mentioned above, the cell production rate reported in this section is an estimation of the absolute number of cells generated per hour in the CVEU or epithelial turnover. In the following sections we investigated whether the reduction of epithelial turnover in the chronic setting was due to decreased number of proliferative cells, increased cell death, and/or a longer division cycle.

### The size of the proliferative compartment is not affected in either acute or chronic inflammation models

We found that Ki-67 protein detection did not provide an accurate estimation of the size of the proliferative compartment in mouse small intestinal epithelium (Figs S3C- S3D). To calculate the number of proliferative cells in the crypt, we, therefore, administrated a combination of vincristine sulphate to halt cell division during metaphase, leaving visible mitotic figures, and BrdU to label cells in S phase. Vincristine arrested the division cycle of all cells entering metaphase for a period of 8 h post-injection (data not shown). BrdU was administered together with the initial vincristine injection and approximately every 2 h thereafter to label cells already in, or entering, S-phase over the following 8 h (Fig. 2c). The total number of BrdU-labelled cells and/or cells in arrested metaphase provided an estimate of the number of proliferative cells along the CVEU (Fig. 2d). This strategy prevented overestimation of proliferative cells by halting cell division of BrdU-labelled cells. Unlike Ki-67 labelled cells, which are detected on the villus (Figs S3C-S3D), BrdU-labelled cells and cells in arrested metaphase were detected only in the crypt region in all our tissue samples (Fig. 2d). Slowly cycling cells in which the length of G1 is <7 h and/or the length of G2 is greater than 8 h may not all be visualised with this technique. However, the reported timescales for the cell cycle phases in mouse crypts^32,33^ indicate that our vincristine/BrdU delivery strategy likely capture most proliferating cells in the crypt.

Our results showed no differences in the size of the proliferative compartment between duodenum and ileum in control animals (Fig. 2e). During TNF-mediated inflammation, we observed a higher number of proliferative cells in acute inflammation in the duodenum, but not the ileum, and no significant effect on the number of proliferative cells was seen in chronic inflammation (Fig. 2e). These results suggest that the reduced cell production observed in chronic inflammation is not associated with a decrease in the number of proliferative cells within the crypt.

### Cell apoptosis increases along the inflamed villus and mainly at the villus tip

**Fig. 3:**
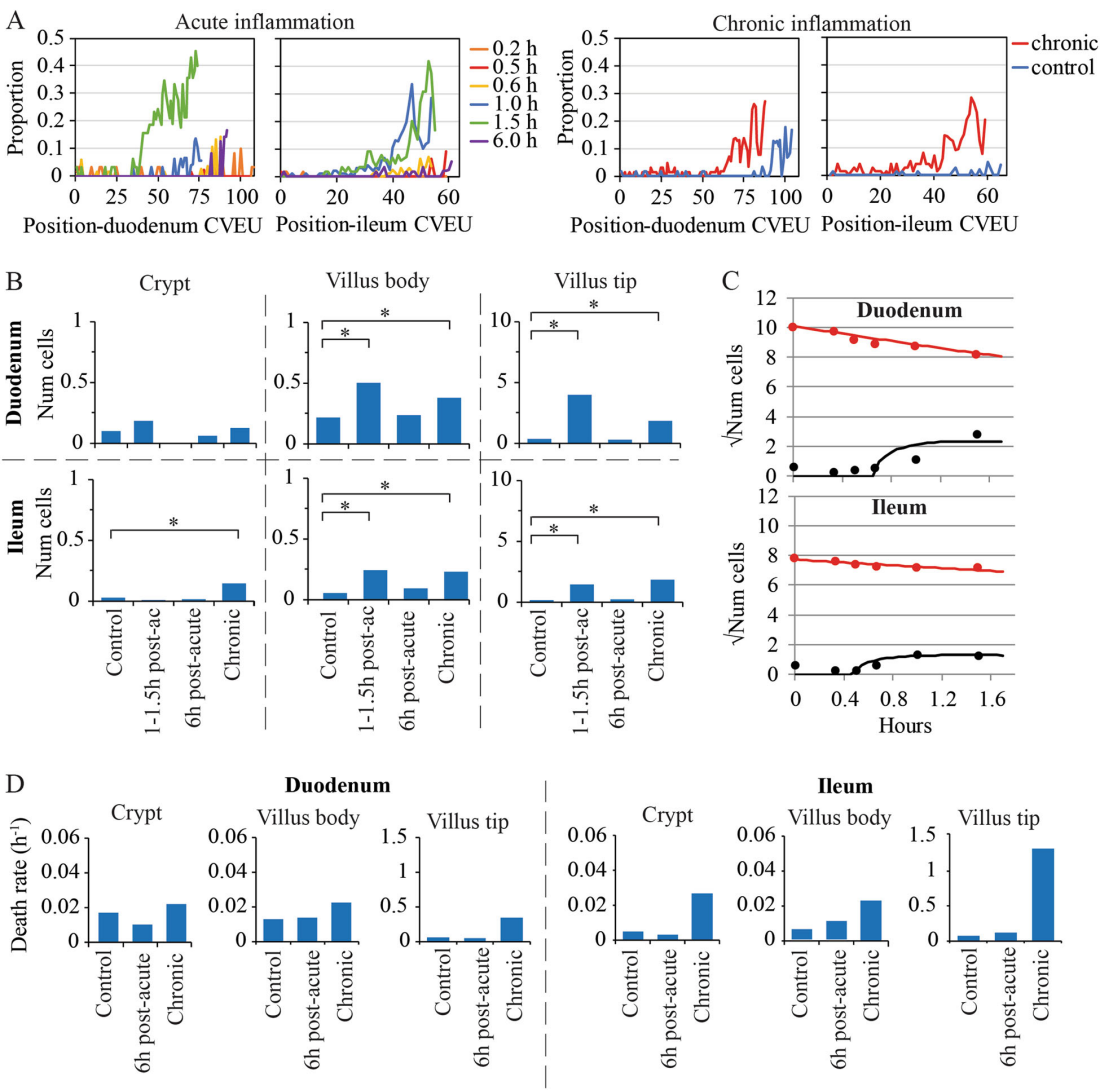
Cell death quantification along the CVEU of chronic and acute inflammation mouse models using TUNEL staining. **a** Proportion of TUNEL-positive cells at each position of the CVEU in duodenum and ileum at several sampling times during acute inflammation and in chronic inflammation and healthy mouse models. **b** Average number of TUNEL-positive cells counted in the crypt, villus body and villus tip in the ileum and duodenum of healthy and inflammation mouse models. Stars denote statistically significant differences. **c** Observed (symbols) and predicted (continuous lines; equations [8–9] in supplementary material) number of TUNEL-positive (black) and -negative (red) cells over time on the CVEU in the duodenum and ileum within the first 1.5 h of the acute inflammation process. **d** Estimates of the cell death rate (h^−1^) in the crypt, villus body and villus tip of healthy mice, chronic inflammation mouse models and during the recovery from acute inflammation (6 h post-acute inflammation)

We observed TUNEL and cleaved-caspase-3 (CC3) positive cells mainly on the villus tips of both inflammation models indicating cell death by apoptosis in agreement with previous reports^8,11^ (Fig. 1c). The numbers of death events increased in both acute and chronic inflammation models with respect to healthy conditions (Fig. 3a). While the majority of dying cells were located at the villus tips, inflammation also increased the number of death events along the villus body (Fig. 3b). An increase in cell death in crypts cannot be clearly concluded from our data as it was significant only in the ileum of the chronic setting (Fig. 3b, Table S2).

We then quantified the temporal dynamics of cell death along the crypt-villus axis. The estimation of death rates enabled the further assessment, as detailed below, of whether the decreased epithelial turnover observed in chronic inflammation is associated with increased cell death. We developed a mathematical model which assumes that death commences when cells become TUNEL-positive and culminates in cells detaching from the epithelium (equations (6–7) in supplementary material). The model considers two cell ‘compartments’, (i) healthy cells and (ii) TUNEL-positive cells, and describes how the number of cells in each compartment changes over time, as healthy cells die, detach, and are lost from the system. The parameters governing these dynamics are the rate of death of healthy cells and the rate of detachment of apoptotic cells (equations (6–7)). Identification of parameter values is described in the supplementary material. Model fits, estimates and errors are given in Fig. 3c, Tables S3 and S4, and Fig. S4.

Cell loss in duodenal and ileal CVEUs (~27 and 8 cells, respectively) during the first hour of acute inflammation (Fig. 3d) is not compensated by cell production (~1.6 and 1.2 cells/h respectively, Fig. 2b, Table S1) resulting in the loss of the villus tips in our acute model (Fig. 1e). Death rates were also increased in the villus body and tip in the chronic setting compared to healthy epithelium, with higher rates in the tip than in the villus body (Table S4 and Fig. 3d). As observed in the acute setting, the high death rate at the villus tip cannot be compensated by cell proliferation and is responsible for the shortening in villus length, which does not recover while circulating TNF levels are elevated. On the other hand, sustained increased cell loss in the villus body is balanced by proliferation, which prevents further reduction of the villus length, but results in reduced epithelial cell turnover in the chronic setting (Fig. 2b).

### Cell division is not affected by chronic TNF-driven injury

**Fig. 4:**
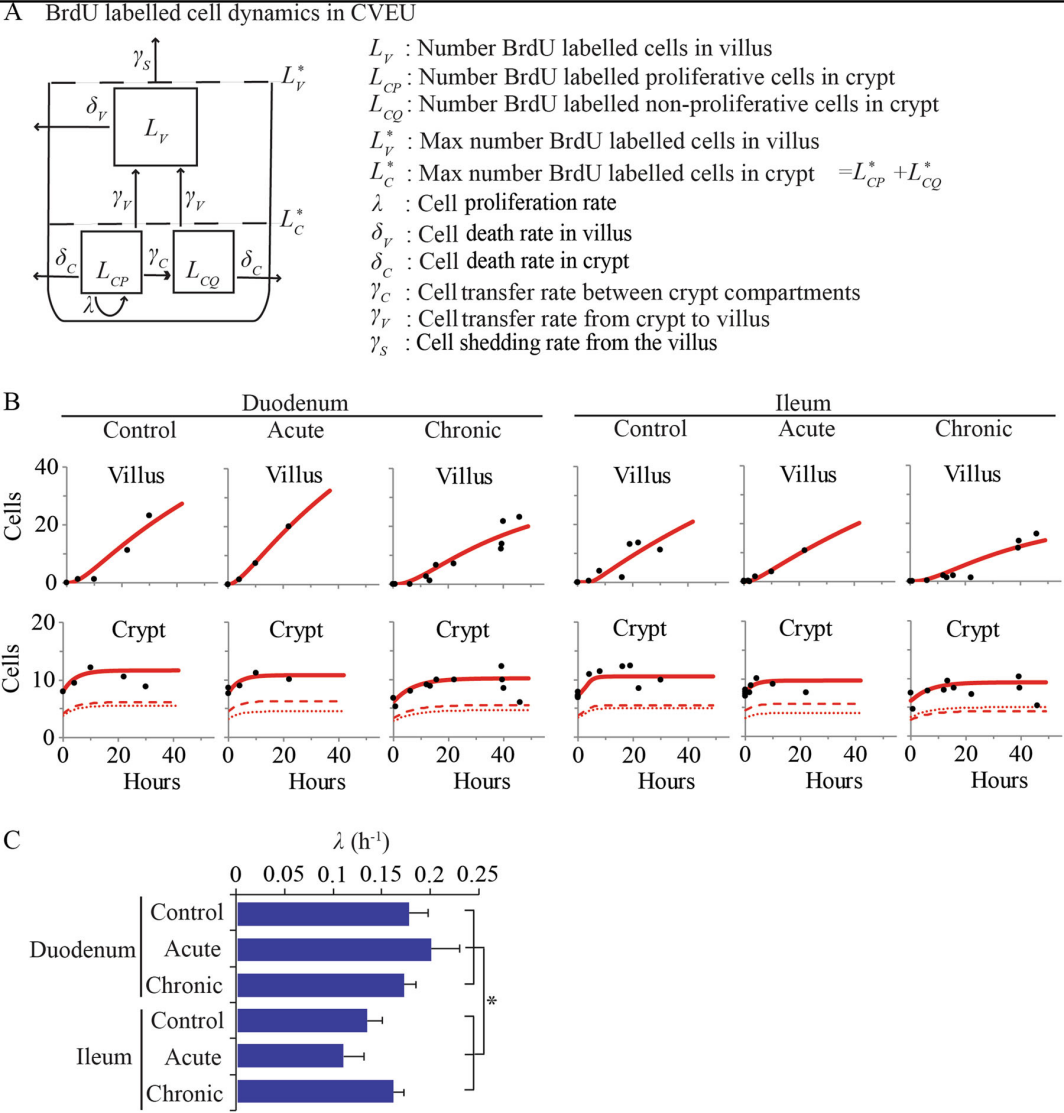
Estimation of the division rate of crypt proliferative cells during chronic and acute inflammation mouse models using BrdU label dynamics and previously gathered information on cell death rates and number of proliferative cells. **a** Schematic of the three-compartment model developed to quantify the temporal dynamics of the BrdU labelling along the CVEU (equations [13–15]). Two of the compartments contain exclusively crypt cells, one with proliferative cells and the other one with non-proliferative cells. The third compartment is the villus compartment, which contains all remaining non-proliferative cells of the CVEU. Following a single BrdU injection, proliferative and non-proliferative BrdU-labelled cells are generated within the crypt and transferred onto the villus once they reach the crypt-villus boundary. Cell death may cause loss of cells from these compartments. Cell shedding of labelled cells from the villus tip starts after labelled cells reach the tip of the villus. **b** Experimental observations (circles) and three-compartment model predictions (lines) of the number of labelled cells over time in the crypt and villus in the duodenum and ileum of control mice and of acute and chronic inflammation mouse models. Dashed and dotted lines represent the predicted number of proliferative and non-proliferative cells, respectively, in the crypt. **c** Comparison of the cell division rate, *λ* (h^−1^), of the crypt proliferative compartment of duodenum and ileum in control mice and in acute and chronic inflammation mouse models. Stars denote statistically significant differences

We next asked whether altered cell cycle duration could be partly responsible, together with increased apoptosis along the villus body, for the reduced cell turnover observed in our chronic setting. To do this, we described the temporal dynamics of BrdU labelling along the CVEU with a three-compartment model^34^. Two compartments comprise crypt cells: one with proliferative cells and the other with non-proliferative cells; the third compartment, or villus compartment, contains all remaining non-proliferative cells of the CVEU (Fig. 4a). We assumed that following a single BrdU injection, proliferative and non-proliferative BrdU-labelled cells are generated within the crypt and transferred onto the villus once they reach the crypt-villus boundary; labelled cells migrate upwards until they are shed from the villus tip. With this model, we evaluated the effect of inflammation on the division rate of the crypt proliferative population. The unambiguous identification of the value of parameters in equations (16–23) was achieved using experimental results from previous sections and BrdU labelling datasets (supplementary material). Fitting diagnosis plots and parameter estimates can be found in Fig. 4b, Fig. S5 and Table S5.

The division rate was faster in duodenum than in ileum (Fig. 4c). We did not observe changes in cell cycle duration during chronic or acute inflammation (Fig. 4c). As mentioned above, this result is inconclusive for the acute setting because the BrdU measurement period exceeds the duration of the acute inflammation process. Our results indicate that during chronic TNF-driven inflammation, a decrease in the overall epithelial turnover is associated with increased cell death in the villus body while cell division or number of proliferative cells in the crypt is not affected. Cell proliferation within the crypt does not respond to compensate for increased cell death on the villus body or to ameliorate the permanent loss of the villus tip.

### The villus tip exhibits elevated intracellular TNF and increased TNFRI expression in response to a pulse of TNF

**Fig. 5:**
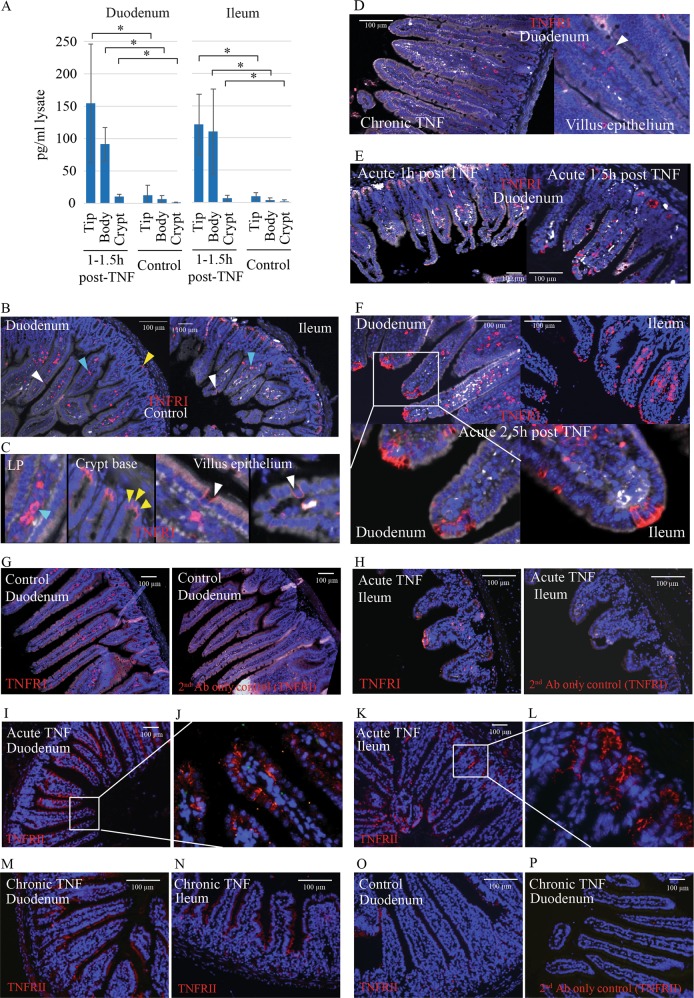
Intraepithelial TNF concentration and TNFRI and TNFRII expression in duodenum and ileum during chronic and acute inflammation. **a** Concentration of TNF in lysates of epithelial cells isolated from the crypt, villus body or villus tip of duodenum and ileum 1–1.5 h after the initiation of the acute inflammatory process vs. control. **b**–**c** Representative images of TNFRI staining in duodenal and ileal sections from healthy mice. TNFRI staining was detected on cells of the lamina propria (blue arrowheads), Paneth cells (yellow arrowheads), and scattered epithelial cells along the villus body resembling goblet or enteroendocrine cells (white arrowheads). **d** TNFRI distribution in chronic inflammation was similar to controls. **e** In acute TNF-treated animals, intense staining was seen in villus tip cells, particularly at sites of tip constriction and shedding at 1–1.5 h post-injection. **f** Clusters of TNFRI-positive cells were observed at the villus tip at later time points after shedding ceased. **g**–**h** Comparison of TNFRI staining and secondary-antibody controls in serial sections of control samples (duodenum) and in acute TNF-treated mice (ileum shown). **i**–**n** Representative images of TNFRII staining in duodenal and ileal sections of acute and chronic TNF models and control duodenum. **o** TNFRII staining in control duodenum. **p** Secondary-antibody-only control for TNFRII staining (duodenum from chronic model shown). TNFRI and TNFRII staining shown in red, nuclei counterstained with Hoescht (blue)^30,32.52–54^

To gain insight into the mechanisms driving apoptotic responses on the villus, we analysed the intracellular concentration of TNF and the expression of TNF receptors I (TNFRI) and II (TNFRII) along different regions of the CVEU. Following a single acute TNF injection, we detected variation in intraepithelial levels of TNF, with an increasing concentration from villus base to tip. Levels of intracellular TNF in villus tips were ~100-fold higher than in healthy mice (Fig. 5a). The detection of high levels of intracellular TNF soon after administration (1–1.5 h) indicates that progressive accumulation of TNF as cells migrate towards the villus tip is not likely to be contributing to intraepithelial gradient formation. It seems more plausible that cells located at the villus tip produce or internalise more TNF than in other CVEU regions.

Immunofluorescence staining for TNFRI (Fig. 5b–h) in healthy tissues did not reveal an expression gradient along the crypt-villus axis, suggesting TNFRI distribution does not explain tip predisposition to TNF-induced death (Fig. 5b). In homoeostasis, TNFRI expression is predominantly located in crypt cells, particularly Paneth resembling cells, in villus cells with secretory cell appearance and in lamina propria cells (Fig. 5c). In chronic inflammation we observed similar patterns of TNFRI expression to homoeostasis (Fig. 5d), however at the peak of acute inflammation (1–1.5 h post-TNF delivery), we found a distinctive expression pattern with TNFRI-positive cells located on the villus tip, at sites of tip constriction and at damaged and shedding villus extremities (Fig. 5e). At later time points, when tip morphology is recovered, clusters of TNFRI-positive cells could still be detected on the villus tip (Fig. 5f), suggesting cells remain sensitised to TNF for some time after intense tip shedding ceases. Dissimilarly, we found TNFRII expression was induced by both acute and chronic TNF delivery (Fig. 5i–p) and was widespread in cells along the CVEU (Fig. 5i–n).

Altogether, these results indicate that cells on the villus tip respond rapidly to TNF by upregulating TNFRI and TNFRII. TNFRI spatial pattern of upregulation is associated with elevated intracellular levels of TNF and cell apoptosis, which results in rapid loss of the tip in acute inflammation. We hypothesise that the maintenance of elevated circulating TNF impairs the recovery of the TNF-sensitive villus tips in our chronic injury model, in which we observe recovered epithelial continuity but reduced villus length.

## Discussion

The integration of labelling techniques with mathematical models here demonstrated a lack of proliferative compensatory response to chronic inflammation. In other reports, proliferative responses to sustained epithelial injury are reported in the colon, but vary with species and injury model. Increased proliferation and apoptosis is reported in DSS colitis in rats^35^, while DSS injury is reported to increase apoptosis, but decrease proliferation in BALB/c mice^36^. Increased cell migration but unaffected proliferation is reported in DSS-treated colon^37^. In a surgical biopsy injury model, increased proliferation was reported exclusively in the colonic crypts adjacent to the site of biopsy^38^.

We found increasing TNF intracellular concentration toward the villus tip in acute inflammation (Fig. 5a). We also saw TNFRI expression localised to tip epithelial cells. It is not clear whether endogenous epithelial-produced TNF or exogenous TNFRI-bound TNF determines tip sensitivity, but it is clear that the response to TNF signalling is more intense in tip cells. Differential expression of TNF-triggered signalling pathways, including MAPK signalling^24,39^ and the detection of pro-apoptotic cleaved villin fragments specifically in tip cells^40^, have also been implicated in TNF-regulated cell death. Upregulation of these factors may be promoted by signalling from tip-localised subsets of sub-epithelial myofibroblasts, which are morphologically and phenotypically distinct from fibroblasts located lower in the villus body^41–44^.

In chronic inflammation, we did not observe tip-localised expression of TNFRI (Fig. 5c), yet caspase and TUNEL-positive staining showed increased tip cell death relative to healthy intestine (Fig. 3b–d). No obvious core contraction or constriction of the villus tip was observed in this setting either. these phenomena are likely to be early events during the onset of chronic inflammation. Thus, TNF-sensitive villus tips were probably lost by the time of sampling and their recovery impaired by the sustained shedding of TNFRI-expressing tip cells. Other authors have also reported different TNFRI expression patterns in response to acute and chronic inflammation^18^.

The TNFRI staining pattern we observe in homoeostasis is in agreement with previous reports describing staining of lamina propria fibroblasts^45^ and ileal L-cells^46^, but does not reproduce conflicting descriptions of more widespread apical^29^ or basolateral^24^ staining of duodenal epithelial cells. Our ileal TNFRI staining following TNF challenge is similar to previous reported patterns in healthy ileum^29^. Disparities likely reflect differences in animal housing and specific antibodies used. We detected widespread TNFRII expression in epithelial cells in our inflammation models in agreement with previous reports^26,47^. The differences we observed in TNFRI and TNFRII staining patterns suggest distinctive location-specific regulation which results in co-localisation of TNFRI, but not of TNFRII, with elevated intracellular TNF and TNF-induced apoptosis.

In chronic TNF injury, we quantified that increased cell death in the villus body and tip was responsible for the slower epithelial turnover and blunted villi, respectively. Together with inflammatory protein expression analyses and mucosal immune cell profiles, these results suggest a repair response to cell damage, which leads to villus re-epithelialisation but fails to recover the original villus length. What causes this state to further destabilise and lead to overt clinical pathology resembling IBD in later weeks^4,14^ remains to be specifically determined. Strong evidence suggests that the microbiota is an essential driver of IBD development^3,4,14^, which may explain why we detect epithelial alterations in both duodenum and ileum in our chronic setting, whereas subsequent IBD lesions are reported to affect the ileum and ileocolonic region^3,4,14^. Of note is that subclinical lesions detected by confocal endomicroscopy are reported in the duodenum of IBD patients^48^.

In summary, we have developed a strategic combination of in vivo models, labelling techniques and mathematical models to assess cell dynamics during acute and chronic injury aiming to understand the response of the epithelium to inflammation. Future detangling of the epithelial response, and the immune and microbial changes during the onset of IBD, is essential to discover new strategies for therapeutic intervention.

## Materials and methods

### Mice

All animal experiments were conducted in strict accordance with the Home Office Animals (Scientific Procedures) Act 1986. Female C57BL/6 mice, aged 10–12 weeks and weighing at least 25 g prior to use in experiments, were housed and maintained in SPF conditions at the University of East Anglia, Norwich, UK in accordance with HO regulations, and all procedures were performed by fully-trained and licenced researchers. Experimental animals were closely monitored and were killed by rising CO_2_ and cervical dislocation, at the time points described in the text, prior to subsequent tissue collection. All animals were regularly monitored for clinical signs; any displaying signs beyond those expected within the moderate limits of the procedures would be immediately sacrificed by the above methods and were not included in experimental data. Osmotic minipumps (Alzet, model 2002, Charles River, Margate, UK) were inserted subcutaneously to anaesthetised animals.

### Inflammation induction and BrdU cell labelling

Transient, acute inflammation was induced by single intraperitoneal injection of recombinant murine TNFα (Peprotech, London, UK) at 0.5 mg/kg. Chronic inflammation was initiated by hydrodynamic tail vein delivery of 10 µg TNF-expressing plasmid (pHEP-TNF a kind gift from C. Gunther, Erlangen, Germany) in a volume of 10% of body weight in Ringer’s Solution (Braun, Germany). Alternatively, low-level TNF (Recombinant murine TNFα, Peprotech, UK) was delivered continuously for 2 weeks at 35 ng/h by subcutaneously implanted osmotic minipump. The minipump delivery setting was used to validate the epithelial turnover results observed in the plasmid mediated setting with similar TNF circulting concentrations. Plasma concentration of TNF was confirmed by specific ELISA (Thermo Fisher Scientific, Waltham, USA) for elevated levels in blood plasma over a minimum of 14 days, and in liver and intestinal tissue lysates post-mortem. The thymine analogue 5-bromo-2-deoxyuridine, BrdU (Sigma-Aldrich, Paisley, UK) was administered at 50 mg/kg body weight by single intraperitoneal injection. In the case of acute inflammation, the experimental ‘time zero’ of the BrdU labelling process was set to 2 h after the single-dose BrdU injection, which we previously demonstrated to be adequate for all mouse models and tissue samples^30^. BrdU was delivered simultaneously with TNF in the acute injury setting. In the chronic inflammation experiments, BrdU time courses were performed once elevated blood TNF levels had been established (Fig. 1a).

### Vincristine delivery

To halt cells in mitosis, 0.5 mg/kg vincristine together with 50 mg/kg BrdU was delivered by single intraperitoneal injection. In the acute model, these two compounds were delivered simultaneously with the injection of TNF, while in the chronic model vincristine and BrdU were administered after ~2 weeks of TNF induction, once elevated plasma levels had been confirmed by ELISA. BrdU was then delivered at 2.33, 4.66 and 7 h post-vincristine injection both in the acute and chronic model (Fig. 2c). Tissues were collected for analysis 8 h after the initial vincristine injection.

### Tissue processing and immunostaining

At sampling time points post BrdU administration, mice were killed and intestinal tracts were removed, dissected, formalin-fixed and paraffin embedded. Transverse sections of duodenum and ileum were prepared at 5 μm and were immunostained for BrdU using biotinylated anti-BrdU antibody (AbCam, Cambridge, UK), Neutravidin-HRP (Thermo Fisher Scientific, Waltham, MA, USA), and diaminobenzidine reaction (DAB, Dako, Glostrup, Denmark). Ki-67-positive cells were detected with Rabbit anti-Ki-67 and goat-anti-rabbit Alexa-568 (AbCam). TNFRI and TNFRII labelling was performed on FFPE sections with rabbit polyclonal antibodies against TNFRI [raised against mouse TNFR1 aa 29–43, GLVPHLGDREKRDSV (AbCam)] or against TNFRII [raised against a synthetic peptide of mouse TNFRII C-terminus aa296–324, QRDAKVPHVPDEKSQDAVGLEQQHLLTTA (Thermo Fisher)] followed by secondary labelling with goat-anti-rabbit-Alexa-568 (Life Technologies). Villus cell apoptosis was confirmed histologically by Caspase-3 [rabbit anti-CC3, (R&D Systems, Minneapolis, USA) and goat-anti-rabbit-HRP, (AbCam)], and general cell death by TUNEL assay (Click-iT TUNEL Alexa Fluor 488, Thermo Fisher Scientific) in FFPE duodenal and ileal sections counterstained with H&E or DAPI. Labelled cell counts for BrdU, Ki-67, caspase 3 and TUNEL were obtained following the format described in ref. ^30^. The numbers of labelled and unlabelled cells were recorded by position, from crypt base to villus tip, for 30–50 individual hemi crypt-villus units per tissue section per mouse, with counts recorded as binary values.

### Epithelial cell isolation

Small intestinal epithelial cells were isolated by removal of whole small intestine which was flushed, opened longitudinally, washed and cut into small (<1 cm) pieces which were then sequentially digested by three rounds of incubation at 37 °C, shaking for 8 min in DPBS/1 mM HEPES/2 mM EDTA. Detached epithelial cells in the resulting supernatants were pelleted and resuspended in IMDM, spun over 40% Percoll layer, aspirated, washed and re-pelleted, prior to preparation of cell lysates for protein analysis, as described below.

### Protein expression analysis

Cell lysates of isolated epithelial cells or whole small intestinal tissue pieces (50 mg) were lysed using Lysing Matrix D ceramic beads (MP Biomedicals, Santa Ana, CA, USA) and Cell Lytic solution (Sigma-Aldrich) containing protease inhibitors. Samples were centrifuged at 13,000 rpm for 10 min at 4 °C. Supernatants were collected and total protein concentration determined using the BCA method (Pierce, Thermo Fisher). Samples were adjusted to normalise total protein concentration before inflammatory protein analysis using Mouse Inflammation Antibody Array (Abcam, Cambridge, UK). Array blots were imaged using chemiluminescence detection and imager (Protein Simple, Oxford, UK). Densitometry analysis of images was performed in Fiji^49^.

### Lamina propria cell isolation and flow cytometry

Lamina propria lymphocytes and myeloid cells were isolated from small intestine using a method adapted from Scott et al.^50^. Briefly, small intestinal pieces (obtained as above) were incubated at 37 °C for 15 min in HBSS/1 mM HEPES/2 mM EDTA, washed four times (shaking, supernatants containing epithelial cells removed) before collagenase/dispase digestion for 1 h at 37 °C. Supernatants were filtered, centrifuged, and pellets resuspended in chilled FACS buffer (DPBS/2% FBS 1 mM EDTA) on ice before antibody staining and flow cytometric analysis.

For flow cytometry identification, gating strategies were based on those previously described^50,51^ and are shown in Fig. S1B. 100,000 cells were analysed per sample. Live/dead discrimination was determined using Zombie UV dye (Biolegend, San Diego, CA, USA). Cells were stained with a panel of immunophenotyping antibodies (Fig. S1C) for 40 min protected from light, at room temperature. Data were acquired with a BD LSRFortessa flow cytometer using BD FACSDiva software (BD Biosciences, San Jose, CA, USA).

### Statistical analysis

The mathematical and statistical models developed to describe temporal cell dynamics in the CVEU are described in supplementary material. Model parameters were estimated using Bayesian inference. Statistical comparison of our experimental groups was carried out by simulating the posterior probability distribution of the difference between model parameters using Markov Chain Monte Carlo methods (MCMC). Differences between groups were considered significant when P{parameter (Group i) > parameter (Group j) | *O*} > 0.95, where *O* represents the dataset used to fit the model.

The significance (*p*-value <0.05) of differences between means of observed quantities in our experimental mouse models was assessed by ANOVA followed by a Tukey multiple comparison test.

Parameter inference and statistical analysis was performed using SAS 9.4

## Supplementary information

Supplemental Material.
