# Supplementary material for: Elevated apoptosis impairs epithelial cell turnover and shortens villi in TNF-driven intestinal inflammation
Source: Cell Death Dis. 2019 Feb 6;10(2):108. doi: 10.1038/s41419-018-1275-5 (PMC6365534; doi:10.1038/s41419-018-1275-5)
Supplement: Supplementary file 1 — Supplemental Material. [file 41419_2018_1275_MOESM1_ESM.pdf]

Supplementary Material

Elevated apoptosis impairs epithelial cell turnover in TNF-driven intestinal inflammation

Parker A[1], Vaux L[1], Patterson AM[1], Modasia A[1], Muraro D[2], Fletcher AG[3,4], Byrne HM[5], Maini PK[5], Watson AJM[6] and Pin C[1,7]*

Theoretical modelling and parameter estimation

Estimation of the absolute cell production rate in the CVEU (epithelial turnover) using BrdU labelling experiments

Based on previous results [1,2], we assumed that cell migration on the villi resembles a conveyor belt at constant velocity determined by cell proliferation in the crypt. Thus, the progression of the labelled front on the villus, X_{LF}, defined as the highest CVEU position with a BrdU-labelled cell, is described by the following expression:

$$\frac{dX_{LF}}{dt} = V , \qquad (1)$$

where V (cells/h) is the cell velocity on the villus and equivalent to the absolute cell production rate of the one-dimensional CVEU (cells/h). This rate results from the balance between proliferation in the crypt and death along the CVEU. We assume that cell migration in the one-dimensional CVEU is a good representation of cell migration in the three-dimensional villus, which received cells from various neighbouring crypts. Supporting this assumption, the genetically labelled progeny of Lgr5+ basal stem cells has been shown to migrate forming longitudinal strips from the base of the crypt to the villus tip [3].

Using our healthy, acute and chronic injury data, we conducted Bayesian parameter inference on this model by Markov chain Monte Carlo (MCMC). To do this, we assumed that the position of the labelled front followed a normal distribution, with standard deviation, *sd*, and mean given by

$$X_{LF}(t) = \sum_{i \in \Omega} (a_i + V_i t) F_i \quad , \tag{2}$$

where Ω is the set of n (=3) experimental conditions or mouse models, which include healthy, acute and chronic inflammation, a_i is the position of the labelled front at time t=0 in condition i, and F_i is an indicator (dummy) variable with value equal to 1 for condition i and 0 otherwise. The likelihood function for O, the set of observed positions of the labelled front in our data, was therefore given by

$$\mathrm{L}(O \mid a_1..a_n, V_1..V_n, sd) = \prod_{h=1}^{m} \mathrm{Normal}(x_h; X_{LF}(t_h) \mid a_1..a_n, V_1..V_n, sd) \tag{3}$$

where 'Normal' denotes the probability density function of a normal distribution with mean X_{LF} and standard deviation sd, x_h is the observed position of the labelled front in the h-th sample, t_h is the time at which the h-th sample is collected, and m is the number of samples collected over time.

The selected prior distributions for the parameters were:

$\pi\ (a_i) \sim$ Uniform (1, 20000) for i =1...n,

where 'Uniform' denotes the probability density function of a uniform distribution with values between 1 and 20000.

$\pi\ (V_i)$ for i =1..n, $\pi\ (sd) \sim$ Gamma (0.001, 0.001)

where 'Gamma' denotes the probability density function of a gamma distribution with mean value equal to 1 and variance 10^3.

Table S1 and Fig S2 show the posterior estimates and the fitting diagnosis plots, respectively, for each experimental dataset.

Analysis of the number of dead cells in the CVEU using TUNEL labelling experiments

We sought to compare the number of dead cells quantified in control conditions with those estimated in the chronic inflammation mouse model and in the acute inflammation mouse model at the peak of the process (1-1.5h post TNF injection) and during recovery (6 h post-TNF injection) in different regions of the CVEU. We assumed that the number of TUNEL-positive cells in the crypt, villus body or villus tip followed a Poisson distribution, whose parameter θ was expressed as a function of the experimental conditions by

$$\theta = \sum_{i \in \Omega} b_i F_i , \qquad (4)$$

where Ω and F_i are as defined above, and b_i denotes the number of dead cells per CVEU in experimental condition *i*. The likelihood function for *O*, the set of observed number of TUNEL-positive cells in our experimental conditions, is therefore given by

$$\mathrm{L}(O \mid b_1..b_n) = \text{Poisson } (d; w\theta \mid b_1..b_n) , \qquad (5)$$

where 'Poisson' denotes the probability density function of the Poisson distribution with parameter $w\theta$ and *d* is the observed number of TUNEL-positive cells in the *w* sampled CVEUs (See Table S2). We selected the conjugate prior distribution for this likelihood:

$\pi\ (b_i) \sim$ Gamma (0.001, 0.001), for $i = 1..n$, where "Gamma" is as defined above. Therefore, the posterior distribution for $b_1..b_n$ was also a gamma distribution with parameters $\sum_{j}^{w} d_j + 0.001$ and $w + 0.001$, which facilitated the exact estimation of the posterior mean, standard deviation and credible intervals for the number of dead cells in each experimental setting (Table S2).

Estimation of cell death rates in the CVEU using TUNEL labelling experiments

To quantify the temporal dynamics of cell death in our experimental models, we developed a mathematical model that describes TUNEL labelling dynamics in epithelial cells. We assumed that epithelial cells that lose viability become TUNEL-positive and detach from the epithelium. We defined *H* as the number of TUNEL-negative (healthy) cells and *D* as the number of TUNEL-positive (dead) cells in the CVEU. Thus, TUNEL labelling dynamics can be described by the following system of ordinary differential equations:

$$\frac{dH}{dt} = -\delta H \qquad (6)$$

$$\frac{dD}{dt} = \delta H - \gamma_D D \qquad (7)$$

where δ is the cell death rate (h^{-1}) and γ_D (h^{-1}) is the rate of detachment of TUNEL-positive cells from the CVEU, both rates being constant over time. With initial values $H(0) = H_0$ and $D(0) = D_0$, which are inferred from the data with the rest of parameters, the solutions of equations (6)-(7) are:

$$H(t) = H_0 e^{-\delta t} \qquad (8)$$

$$D(t) = D_0 e^{-\gamma_D t} + \frac{H_0}{\frac{\gamma_D}{\delta} - 1}(e^{-\delta t} - e^{-\gamma_D t}) \quad . \tag{9}$$

Parameter estimation was performed using Bayesian inference. We assumed that both the number of TUNEL positive and negative cells in CVEU followed a Poisson distribution with parameters equal to *H* (*t*) and *D* (*t*) described in equations (8) and (9), respectively, which were dependant on experimental conditions and collected in an unique expression, *θ*, as follows:

$$\theta(t) = \sum_{i \in \Omega} (H_i(t)\xi_H + D_i(t)(1 - \xi_H))F_i , \tag{10}$$

where Ω and F_i are as defined above, H_i and D_i correspond to the expressions in equations (8)-(9) for experimental conditions (or mouse model) *i*, ξ_H is an indicator variable with value equal to 1 for TUNEL-negative (healthy) cells, and to 0 otherwise.

The likelihood function for *O*, the set of observed TUNEL positive and negative cells, was therefore given by:

$$\begin{aligned} &\mathrm{L}(O \mid D_{0,1}..D_{0,n}, H_{0,1}..H_{0,n}, \delta_1..\delta_n, \gamma_1..\gamma_n) = \\ &\prod_{h=1}^{m} \mathrm{Poisson}(h_h, d_h; w_h \cdot \theta(t_h) \mid D_{0,1}..D_{0,n}, H_{0,1}..H_{0,n}, \delta_1..\delta_n, \gamma_1..\gamma_n) \end{aligned} , \tag{11}$$

where h_h and d_h are the observed number of TUNEL negative and positive cells, respectively, in the w_h samples (CVEUs) collected at the t_h sampling time and *m* is the number of time points.

The selected prior distributions for the parameters were:

π ($H_{0,i}$), π ($D_{0,i}$) ~ Uniform (1, 20000) for $i = 1..n$ and

π (δ_i), π ($\gamma_{D,i}$) ~ Gamma (0.001, 0.001) for $i = 1..n$,

which are as defined above.

The model (10) was fitted to the TUNEL labelling dataset obtained at several time points in acute inflammation. Table S3 and Fig S4 show the fitting diagnoses plots and parameter estimates obtained for duodenum and ileum during acute inflammation. The steady number of TUNEL positive and negative cells observed in healthy and chronic inflammation is not suited for the unambiguous identification of the model parameter values in equation (10). The invariant numbers of TUNEL positive and negative cells in these mouse models result from the equilibrium between cells turning TUNEL-positive and their detachment from the

epithelium. Alternatively, we estimated the rate of cell death in healthy and chronic inflammation using the relationship between the number of TUNEL positive, D_{ss}, and negative, H_{ss}, cells and the model parameters in steady state derived from equations (6)-(7). This relationship has the following form:

$$\frac{H_{ss}}{D_{ss}} = \frac{\gamma_D}{\delta} \quad . \tag{12}$$

In addition, we assumed that the rate of detachment of TUNEL-positive cells, γ_D, was not affected by the experimental conditions used in this work. This implied that the detachment rate estimated in acute inflammation conditions was considered a good estimate for all our experimental conditions. This assumption was partially supported by the estimation of similar values for the detachment rate in ileum and duodenum which, in contrast, exhibited different death rates during acute inflammation (Table S3). The steady-state number of TUNEL-positive and negative cells are reported in Fig 3B and Table S4 for each experimental condition.

Estimation of cell division rate by modelling BrdU cell labelling dynamics across a CVEU described by three-compartments.

We used the three-compartment model developed in our previous work [4] to describe BrdU labelling dynamics in the CVEU. This model comprises three cell compartments. Two of the compartments represent exclusively crypt cells: one with proliferative crypt cells and the other with non-proliferative or quiescent crypt cells. The third compartment represents the villus, which contains all remaining non-proliferative cells of the CVEU. The model describes how production of cells in the CVEU results from the balance between the size of the crypt proliferative compartment, the duration of the division cycle and cell death along the CVEU and it is compensated by cell shedding from the villus tip (Fig 4A).

We have modelled the propagation of BrdU labelling across these three compartments after a pulse of BrdU (see Fig 4A). Following BrdU injection, proliferative and non-proliferative BrdU-labelled cells are generated within the crypt and transferred onto the villus once labelled cells reach the crypt-villus boundary, which is modelled with a threshold for the number of labelled cells within the crypt. We assumed a common value for the rate of cell death in the two crypt compartments, proliferative and non-proliferative, but this rate might differ for villus cells. Cell shedding from the villus is initiated when labelled cells reach the villus tip or, equivalently, the number of labelled cells in the villus reaches a threshold value. As in our previous work [1,4], we restrict our attention to the period before the cell BrdU content has been diluted below the detection limit, which is observed after 4-5 generations[5].

With these assumptions, the temporal dynamics of our model satisfy the following system of ordinary differential equations:

$$\frac{dL_{CP}}{dt} = \lambda L_{CP} - \gamma_C L_{CP} - \delta_C L_{CP} - \gamma_V (L_{CP} - L^*_{CP}) H(L_{CP} - L^*_{CP}) \quad (13)$$

$$\frac{dL_{CQ}}{dt} = \gamma_C L_{CP} - \delta_C L_{CQ} - \gamma_V (L_{CQ} - L^*_{CQ}) H(L_{CQ} - L^*_{CQ}) \quad (14)$$

$$\begin{aligned}\frac{dL_V}{dt} = {} & \gamma_V (L_{CQ} - L^*_{CQ}) H(L_{CQ} - L^*_{CQ}) + \gamma_V (L_{CP} - L^*_{CP}) H(L_{CP} - L^*_{CP}) - \\ & \delta_V L_V - \gamma_S (L_V - L^*_V) H(L_V - L^*_V)\end{aligned} \quad (15)$$

where $L_{CP}(t)$ and $L_{CQ}(t)$ denote the numbers of proliferative and non-proliferative BrdU-labelled cells in the crypt, respectively, and $L_V(t)$ denotes the number of BrdU-labelled cells in the villus at time t. Here λ(h^{-1}) ≥ 0 is the division rate of crypt proliferative cells, δ_C (h^{-1}) ≥ 0 and δ_V (h^{-1}) ≥ 0 are the death rates of crypt and villus cells, respectively, γ_C (h^{-1}) ≥ 0 and γ_V (h^{-1}) ≥ 0 are the rates of cell transfer between the proliferative and non-proliferative compartment within the crypt and between the crypt compartments and the villus, respectively, γ_S (h^{-1}) ≥ 0 is the rate of cell shedding from the villus tip, and $H(x) = \begin{vmatrix} 0 & \text{if } x \leq 0 \\ 1 & \text{if } x > 0 \end{vmatrix}$ is the Heaviside step function.

We impose initial conditions $L_{CP}(t_0) = L_{CP0}$, $L_{CQ}(t_0) = L_{CQ0}$ and $L_V(t_0) = L_{V0}$ and denote by t_1 and t_2, such that $t_0 \leq t_1 \leq t_2$, the times at which the number of labelled cells within the crypt and on the villus reach the threshold values $L_C{}^* = L_{CP}{}^* + L_{CQ}{}^*$, and $L_V{}^*$, respectively, with $L_{CP0} < L_{CP}{}^*$, $L_{CQ0} < L_{CQ}{}^*$ and $L_{V0} < L_V{}^*$. Here, we report the explicit solution of these equations for the particular case when $\lambda - \gamma_C \neq \delta_C + \gamma_V \neq 0$ and $\delta_C + \gamma_V \neq \delta_V \neq 0$ for the intervals $t_0 \leq t < t_1$ and $t_1 \leq t < t_2$, i.e. before labelled cells reach the villus tip. We define $\xi = \lambda - \gamma_C - \delta_C - \gamma_V$. These solutions are:

$t_0 \leq t < t_1$:

$$L_{CP} = L_{CP0} e^{(\lambda - \gamma_C - \delta_C)(t - t_0)} \quad (16)$$

$$L_{CQ} = \frac{\gamma_C}{(\lambda - \gamma_C)} L_{CP0} \left[e^{(\lambda - \gamma_C - \delta_C)(t - t_0)} - e^{-\delta_C (t - t_0)} \right] + L_{CQ0} e^{-\delta_C (t - t_0)} \quad (17)$$

$$L_V = L_{V0} e^{-\delta_V (t - t_0)} \quad (18)$$

$t_1 \leq t < t_2$:

$$L_{CP}=\frac{L_{CP}^{*}}{\xi}\left[(\xi+\gamma_V)e^{\xi(t-t_1)}-\gamma_V\right] \quad , \tag{19}$$

$$L_{CQ}=\frac{L_{CQ}^{*}\gamma_V}{\delta_C+\gamma_V}+\frac{L_{CP}^{*}\gamma_C}{\xi}\left(\frac{\xi+\gamma_V}{\lambda-\gamma_C}e^{\xi(t-t_1)}-\frac{\gamma_V}{\delta_C+\gamma_V}\right)+K_1e^{-(\delta_C+\gamma_V)(t-t_1)}, \tag{20}$$

$$L_V=L_{CP}^{*}\frac{\gamma_V^2}{\delta_V\xi}\left[\frac{\delta_V\lambda(\xi+\gamma_V)e^{\xi(t-t_1)}}{\gamma_V(\lambda-\gamma_C)(\xi+\delta_V)}+\frac{\xi-\lambda}{\delta_C+\gamma_V}-\frac{\xi}{\gamma_V}\right]-L_{CQ}^{*}\frac{\gamma_V\delta_C}{\delta_V(\delta_C+\gamma_V)}+\frac{\gamma_V K_1}{\delta_V-\delta_C-\gamma_V}e^{-(\delta_C+\gamma_V)(t-t_1)}+K_2e^{-\delta_V(t-t_1)} \tag{21}$$

where

$$K_1=L_{CQ}^{*}\frac{\delta_C}{\delta_C+\gamma_V}+L_{CP}^{*}\frac{\gamma_C}{\xi}\left(\frac{\gamma_V}{\delta_C+\gamma_V}-\frac{\xi+\gamma_V}{\lambda-\gamma_C}\right)L_{CP}^{*} \tag{22}$$

and

$$K_2=L_V(t_1)+L_{CP}^{*}\frac{\gamma_V{}^2}{\delta_V\xi}\left[\frac{\xi}{\delta_V}-\frac{\xi-\lambda}{(\delta_C+\gamma_V)}-\frac{\delta_V\lambda(\xi+\gamma_V)}{\gamma_V(\lambda-\gamma_C)(\xi+\delta_V)}\right]+L_{CQ}^{*}\frac{\gamma_V\delta_C}{\delta_V(\delta_C+\gamma_V)}-\frac{\gamma_V K_1}{\delta_V-\delta_C-\gamma_V} \tag{23}$$

The values of the parameters in equations (16)-(23) were identified by using BrdU datasets as well as the results on the size of the proliferative compartment and on cell death along the CVEU reported in this work.

Our experimental period was restricted to the interval $t_0 \leq t < t_2$, before labelled cells reach the villus tip so that the shedding rate of labelled cells from the tip during this period was γ_s=0. Both t_0, initial time, and L_{V0}, initial number of labelled cells on the villus, were given fixed values: $t_0 = L_{V0} = 0$. The values for the parameters L_{CP0} and L_{CQ0}, initial number of proliferative and non-proliferative labelled cells in the crypt, respectively, were estimated from the average number of labelled cells in the crypt observed 2h post-BrdU injection. BrdU labelling does not enable the distinction between proliferative and non-proliferative descendants. However, the estimation of these two parameters is feasible under the assumption that the ratio between the number of proliferative, *CP*, and non-proliferative, *CQ*, cells in the crypt is constant in our experimental conditions, which is supported by our previous results (Fig 2E) and that BrdU labelling does not affect the rate at which cells enter the quiescent state. Under these assumptions, we have that $\frac{L_{CP0}}{L_{CQ0}}=\frac{CP}{CQ}$. Estimates for *CP* were obtained in this work by the combined administration of BrdU with Vincristine and are reported in Fig 2E. The

size of the crypt compartment, $CP + CQ$, was given a fixed value equal to 20 for all cases, which is the average number of cells from the base to the mouth of the crypt.

The unambiguous identification of the values of the other parameters in equations (16)-(23) involved the use of results obtained in previous sections. As discussed above, BrdU datasets do not enable the robust joint estimation of γ_C, the rate at which quiescent cells are generated in the crypt, and λ, the growth rate of the proliferative population. To circumvent the lack of information in these datasets, we derived the relationship between the steady state number of labelled cells and the model parameters from equations (13)-(14), which has the form:

$$\gamma_C = (\delta_C + \gamma_V)\frac{L_{CQ}^{stat}}{L_{CP}^{stat}}\frac{L_{CP}^{*}}{L_{CQ}^{*} + L_{CP}^{*}} + (\lambda - \delta_C - \gamma_V)\frac{L_{CQ}^{*}}{L_{CQ}^{*} + L_{CP}^{*}} \qquad (24)$$

As described above, we assumed that the ratio between the number of proliferative, CP, and non-proliferative, CQ, cells in the crypt is assumed to be constant under our experimental conditions and not affected by BrdU uptake and given the structure of our model we have that $\frac{L_{CP0}}{L_{CQ0}} = \frac{L_{CP}^{*}}{L_{CQ}^{*}} = \frac{L_{CP}^{stat}}{L_{CQ}^{stat}} = \frac{CP}{CQ}$. Under these assumptions, equation (24) can be expressed as:

$$\gamma_C = \lambda \frac{CQ}{CP + CQ} \quad , \qquad (25)$$

Estimates for CP and CQ were obtained as described above. The replacement of γ_C in equations (16-23) by the expression in equation (25) and the use of the estimates of the death rates, δ_C and δ_V, reported in Table S4, facilitated the unambiguous identification of the values of the rest of the model parameters in equations (16)-(23). These parameters were λ (growth rate of the crypt proliferative population), γ_V (cell transfer rate from the crypt to the villus) and t_1 (time for cell transfer from crypt to villus to start), and were estimated by fitting equations (16)-(23) to the observed number of BrdU labelled cells in the crypt, which comprised the first 20 positions of the CVEU, and villus. Table S5, shows the solutions obtained by non-linear regression methods (Gauss-Newton iterative method).

To compare statistically the value of the parameter λ, cell division rate, between our mouse models, we conducted Bayesian inference to estimate the posterior distribution of λ using MCMC methods. As in previous sections, we assumed that the number of labelled cells

in both the crypt and the villus had a Poisson distribution with parameter θ, which was dependent on time and experimental conditions as follows:

$$\theta(t) = \sum_{i \in \Omega} (L_{C,i}(t)\xi_C + L_{V,i}(t)(\xi_C - 1))F_i \tag{26}$$

where Ω and F_i are as defined above; $L_{C,i} = L_{CP,i} + L_{CQ,i}$ and $L_{V,i}$ correspond to the number of BrdU-labelled cells in the crypt and villus, respectively, described in equations (16)-(23) for experimental condition (mouse model) i; ξ_C is an indicator variable with value equal to 1 for crypt cells, or, otherwise 0.

The distribution function for the likelihood of the dataset was:

$$\mathrm{L}(O \mid \lambda_1..\lambda_n) = \prod_{h=1}^{m} \mathrm{Poisson}(l_{C,h}, l_{V,h}; w_h \cdot \theta(t_h) \mid \lambda_1..\lambda_n) \ , \tag{27}$$

where $l_{C,h}$ and $l_{V,h}$ are the observed number of labeled cells in the crypt and villus, respectively, in the w_h samples collected at the t_h sampling time and m is the number of time points. We selected non-informative prior distributions for the parameters: $\pi(\lambda_i) \sim$ Gamma (0.001, 0.001), which is defined above.

Fitting diagnosis plots and posterior estimates can be found in Fig S5 and Table S5, respectively.

References

1 Parker, A. *et al.* Cell proliferation within small intestinal crypts is the principal driving force for cell migration on villi. *FASEB J* **31**, 636-649, doi:10.1096/fj.201601002 (2017).

2 Maclaren, O. J. *et al.* A hierarchical Bayesian model for understanding the spatiotemporal dynamics of the intestinal epithelium. *PLoS Comput Biol* **13**, e1005688, doi:10.1371/journal.pcbi.1005688 (2017).

3 Barker, N. *et al.* Identification of stem cells in small intestine and colon by marker gene Lgr5. *Nature* **449**, 1003-U1001 (2007).

4 Muraro, D. *et al.* Chronic TNFalpha-driven injury delays cell migration to villi in the intestinal epithelium. *J R Soc Interface* **15**, doi:10.1098/rsif.2018.0037 (2018).

5 Wilson, A. *et al.* Hematopoietic stem cells reversibly switch from dormancy to self-renewal during homeostasis and repair. *Cell* **135**, 1118-1129, doi:10.1016/j.cell.2008.10.048 (2008).

Table S1. Posterior mean, error and credible intervals estimated for each model parameter of equation (2) by MCMC[1].

Experimental conditions		Model parameter		Estimates			
Tissue	Treatment	Description	Code	Mean	Standard deviation	95% Highest posterior density interval	
Duodenum	None	Position LF[2] at t=0	a_WD	17.51	2.22	13.40	22.12
		Prod. rate (cells/h)	V_WD	1.56	0.13	1.31	1.83
	Acute inflammation	Position LF at t=0	a_WD_Ta	21.00	2.45	16.27	25.83
		Prod. rate (cells/h)	V_WD_Ta	1.63	0.27	1.08	2.15
	Chronic inflammation	Position LF at t=0	a_WD_Tc	14.22	3.18	8.09	20.43
		Prod. rate (cells/h)	V_WD_Tc	1.27	0.09	1.10	1.45
Ileum	None	Position LF at t=0	a_WI	19.31	1.15	17.01	21.52
		Prod. rate (cells/h)	V_WI	1.26	0.08	1.09	1.43
	Acute inflammation	Position LF at t=0	a_WI_Ta	21.09	1.03	19.10	23.12
		Prod. rate (cells/h)	V_WI_Ta	1.10	0.10	0.91	1.31
	Chronic inflammation	Position LF at t=0	a_WI_Tc	14.85	1.77	11.17	18.13
		Prod. rate (cells/h)	V_WI_Tc	0.96	0.06	0.84	1.09
		Error position LF	sd	6.76	0.89	5.19	8.55

[1] Markov chain Monte Carlo methods. Diagnosis plots can be found in Supplementary Figure S2.

[2] Labelled front

Table S2. TUNEL positive cell counts and posterior mean, error and credible intervals estimated for the number of dead cells per CVEU.

Experimental conditions			Data		Parameter estimates				
Tissue	CVEU region	Treatment	Counted CVEUs -*w*	TUNEL+ cells -*d*	Parameter code	Mean[1]	Standard deviation	95% Posterior credible interval	
Duodenum	Crypt	None	50	5	b_Ctrl	0.100	0.045	0.032	0.205
		1-1.5h post-acute	32	6	b_Ta1h	0.188	0.077	0.069	0.365
		6h post-acute	17	1	b_Ta6h	0.059	0.059	0.001	0.217
		Chronic	63	8	b_Tc	0.127	0.045	0.055	0.229
	Villus body	None	50	11	b_Ctrl	0.220	0.066	0.110	0.368
		1-1.5h post-acute	32	16	b_Ta1h	0.500	0.125	0.286	0.773
		6h post-acute	17	4	b_Ta6h	0.235	0.118	0.064	0.516
		Chronic	63	24	b_Tc	0.381	0.078	0.244	0.548
	Villus tip	None	55	24	b_Ctrl	0.436	0.089	0.280	0.627
		1-1.5h post-acute	32	127	b_Ta1h	3.969	0.352	3.308	4.688
		6h post-acute	17	5	b_Ta6h	0.294	0.132	0.096	0.602
		Chronic	63	115	b_Tc	1.825	0.170	1.507	2.174
Ileum	Crypt	None	74	2	b_Ctrl	0.027	0.019	0.003	0.075
		1-1.5h post-acute	100	1	b_Ta1h	0.010	0.010	0.000	0.037
		6h post-acute	61	1	b_Ta6h	0.016	0.016	0.000	0.061
		Chronic	76	11	b_Tc	0.145	0.044	0.072	0.242
	Villus body	None	74	4	b_Ctrl	0.054	0.027	0.015	0.118
		1-1.5h post-acute	100	24	b_Ta1h	0.240	0.049	0.154	0.345
		6h post-acute	62	6	b_Ta6h	0.097	0.040	0.036	0.188
		Chronic	76	19	b_Tc	0.250	0.057	0.151	0.374
	Villus tip	None	74	10	b_Ctrl	0.135	0.043	0.065	0.231
		1-1.5h post-acute	100	153	b_Ta1h	1.530	0.124	1.297	1.782
		6h post-acute	63	14	b_Ta6h	0.222	0.059	0.122	0.353
		Chronic	76	134	b_Tc	1.763	0.152	1.477	2.074

Table S3. Posterior mean, error and credible intervals estimated for each model parameter of Equation 10, which describes cell death kinetics, fitted by MCMC[1] methods to the TUNEL labelling dataset obtained from the acute inflammation mouse model.

Model parameter			Estimate			
Tissue	Description	Code	Mean	Standard deviation	95% Highest posterior density interval	
Duodenum	Death rate (h^{-1})	δ	0.27	0.02	0.24	0.30
	Detachment rate (h^{-1})	γ_D	3.42	0.28	2.92	4.01
	Dead cells at $t = 0.66$	D_0	0.12	0.06	0.00	0.23
	Healthy cells at $t = 0$	H_0	97.45	1.10	95.21	99.56
Ileum	Death rate (h^{-1})	δ	0.13	0.01	0.10	0.16
	Detachment rate (h^{-1})	γ_D	3.93	0.29	3.37	4.52
	Dead cells at $t = 0.5$	D_0	0.18	0.02	0.14	0.22
	Healthy cells at $t = 0$	H_0	59.35	0.67	58.06	60.67

[1] Markov chain Monte Carlo methods. Diagnosis plots can be found in Supplementary Figure S4.

Table S4. Estimates of cell death rates in control, recovering after acute inflammation and chronic inflammation mouse models using equation (12).

Tissue	CVEU region	Treatment	TUNEL posterior cells $-D_{ss}$	TUNEL negative cells $-H_{ss}$	Cell death rate (h^{-1}) $-\delta$
Duodenum	Crypt	Control	0.100	19.90	0.018
		6h post-acute inflammation	0.059	19.94	0.011
		Chronic inflammation	0.127	19.87	0.024
	Villus body	Control	0.220	58.78	0.014
		6h post-acute inflammation	0.235	58.76	0.015
		Chronic inflammation	0.381	58.62	0.024
	Villus tip	Control	0.345	19.65	0.065
		6h post-acute inflammation	0.294	19.71	0.055
		Chronic inflammation	1.825	18.17	0.370
Ileum	Crypt	Control	0.027	19.97	0.005
		6h post-acute inflammation	0.016	19.98	0.003
		Chronic inflammation	0.145	19.86	0.027
	Villus body	Control	0.054	34.95	0.006
		6h post-acute inflammation	0.097	34.90	0.010
		Chronic inflammation	0.229	34.77	0.024
	Villus tip	Control	0.135	6.86	0.072
		6h post-acute inflammation	0.222	6.78	0.121
		Chronic inflammation	1.843	5.16	1.315

Table S5. Mean, error and intervals estimated for model parameters in equations (16-23) using BrdU labelling datasets

Experimental conditions		Model parameter	Estimates				[1]Estimation method
Tissue	Treatment		Mean	Standard deviation	95% density interval		
Duodenum	None	[2] λ	0.181	0.017	0.148	0.214	MCMC
		[3] γ_V	0.308	0.422	-0.689	1.305	nlin
		[4] t_I	0.767	5.399	-12.000	13.533	nlin
	Acute inflammation	λ	0.205	0.025	0.159	0.256	MCMC
		γ_V	0.375	0.136	0.053	0.696	nlin
		t_I	-0.214	1.196	-3.041	2.613	nlin
	Chronic inflammation	λ	0.177	0.009	0.160	0.194	MCMC
		γ_V	0.214	0.160	-0.121	0.549	nlin
		t_I	1.040	4.973	-9.370	11.449	nlin
Ileum	None	λ	0.137	0.014	0.110	0.165	MCMC
		γ_V	0.625	4.101	-7.958	9.208	nlin
		t_I	3.881	11.478	-20.142	27.903	nlin
	Acute inflammation	λ	0.111	0.021	0.073	0.154	MCMC
		γ_V	0.385	0.239	-0.128	0.898	nlin
		t_I	0.588	1.352	-2.312	3.487	nlin
	Chronic inflammation	λ	0.165	0.101	0.146	0.185	MCMC
		γ_V	0.207	0.220	-0.257	0.671	nlin
		t_I	1.549	6.310	-11.763	14.862	nlin

[1]Non-linear regression (nlin) or Markov chain Monte Carlo methods (MCMC). Diagnosis plots can be found in Supplementary Figure S5.

[2] Proliferation rate (h^{-1}) of crypt proliferative cell population.

[3]Cell transfer rate from crypt to villus (h^{-1}).

[4]Time (h) for labelled cells to reach threshold for cell transfer to start from crypt to villus.

A

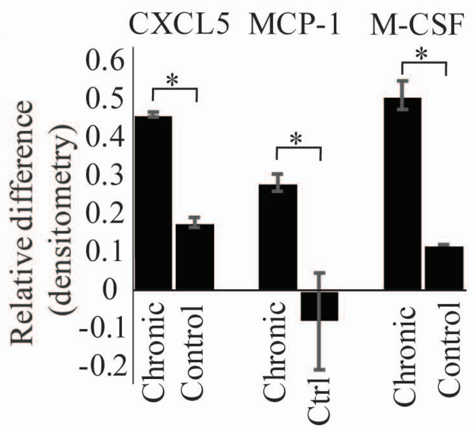

B Single viable cells: SSC-A FSCA debris exclusion; FSC-A FSC-H single cells, SSC-A, Zombie UV live/dead, CD45+

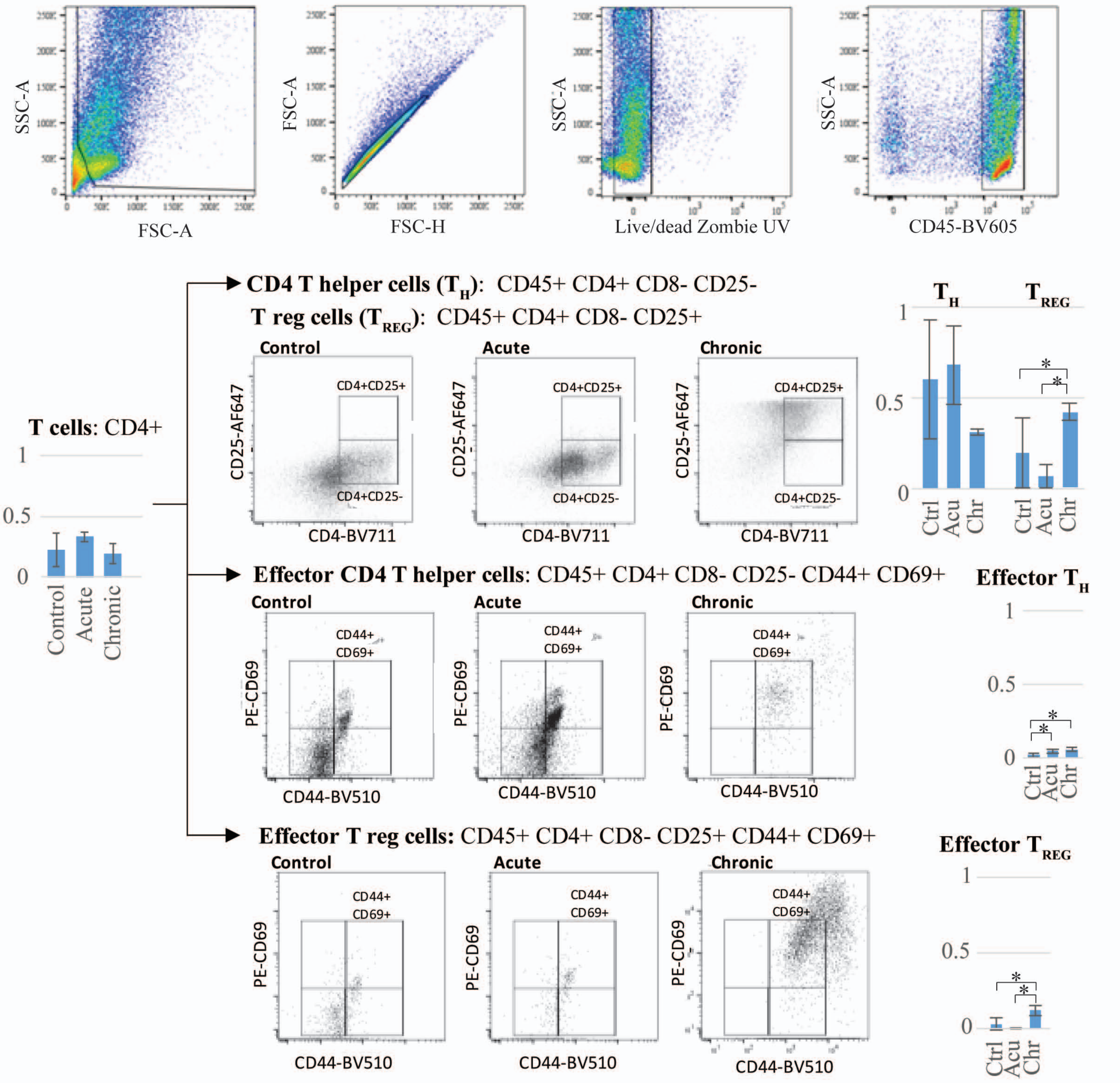

C **Antibody panel for immunophenotyping by flow cytometry**

Antibody	Clone	Conjugate	Company	Catalog #
CD4	RM45	BV711	Biolegend	100550
CD45	30-F11	BV605	Biolegend	103140
CD44	IM7	BV510	Biolegend	103043
CD69	H1.2F3	PE	Biolegend	104508
CD8a	53-6.7	PE-Dazzle	Biolegend	100762
CD25	PC61	AF647	Biolegend	102012
IA/IE	M5/114.152	AF700	Biolegend	107622
Zombie UV (live/dead)	-	UV	Biolegend	423108

Figure S1. Immunological response in inflammation mouse models. A) Relative change in inflammatory response proteins in chronic inflammation. Densitometry analysis of an antibody array membrane blot from complete small intestinal lysates. B) Strategy used for flow cytometry analysis of isolated intestinal mucosal cells. Two-dimensional graphs show the channels used to differentiate isolated mucosal cells with the differentiated populations gated in black. Column plots show the average and error of the proportions of the gated cell populations for each mouse model. C) Antibodies used in flow cytometry analysis of isolated mucosal cells. Stars denote statistical significance difference between mouse models.

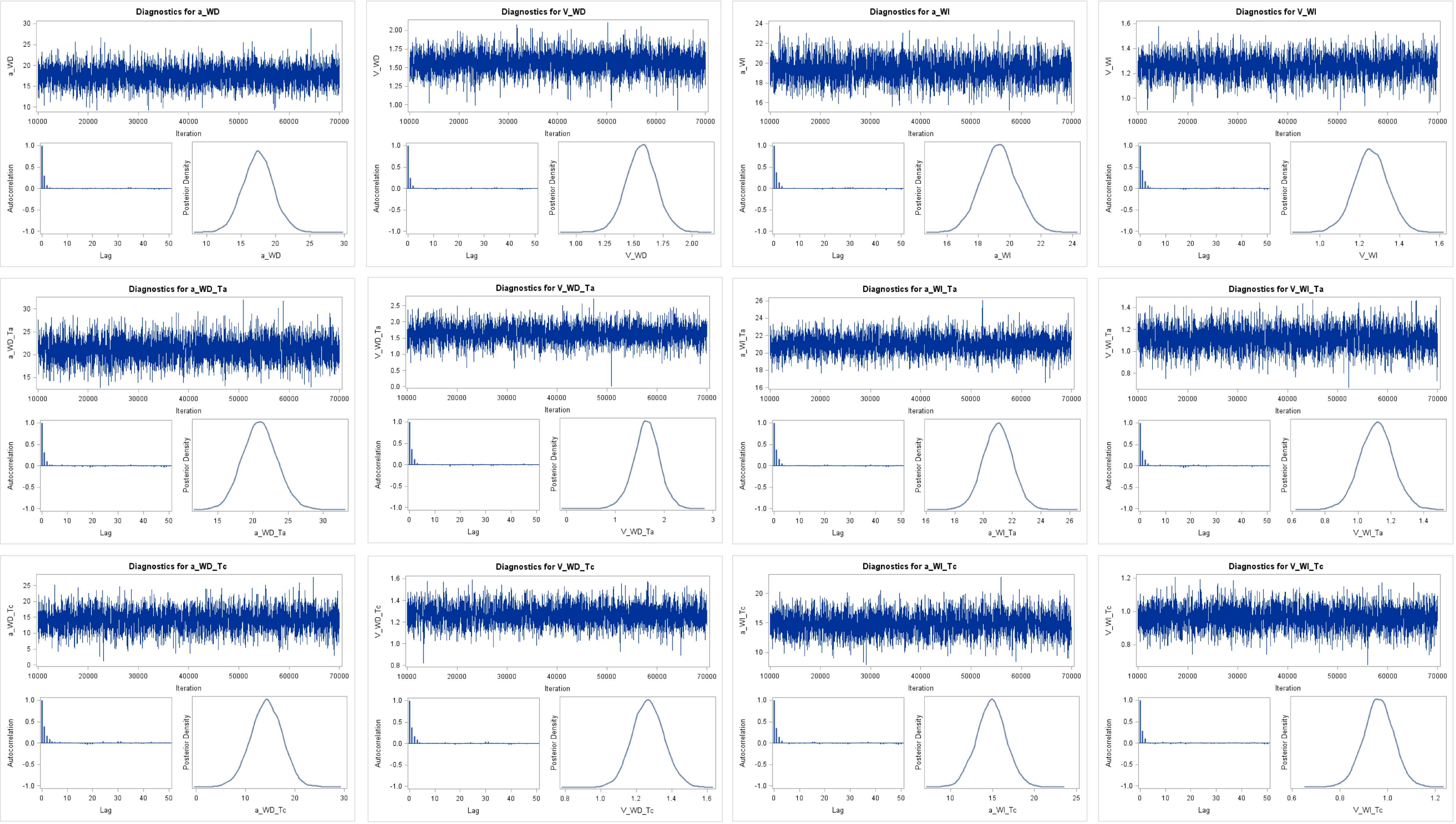

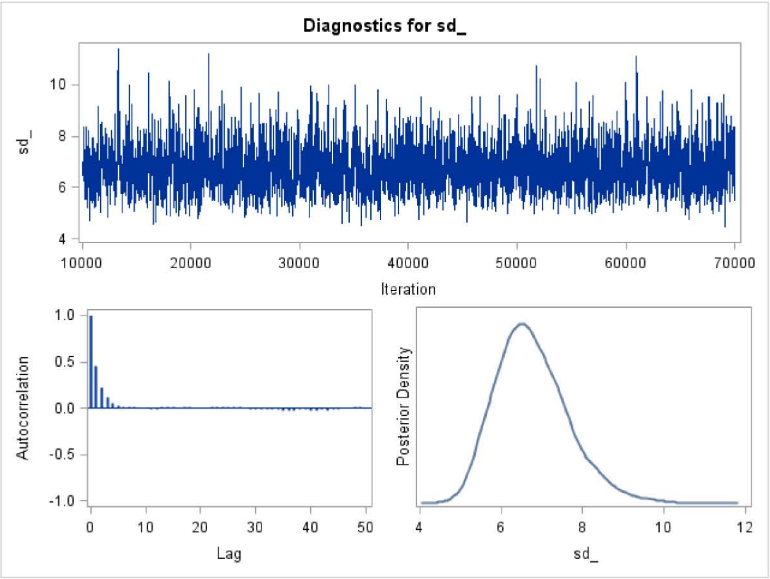

Figure S2. MCMC diagnosis plots for the estimates of the parameters of model in equation (2). The description of the parameters can be found in Table S1. Panels show three plots for each parameter: traces or parameter value estimated in each step of the MCMC (top); autocorrelation functions for the estimates of the parameter (bottom left) and the parameter posterior distribution (bottom right).

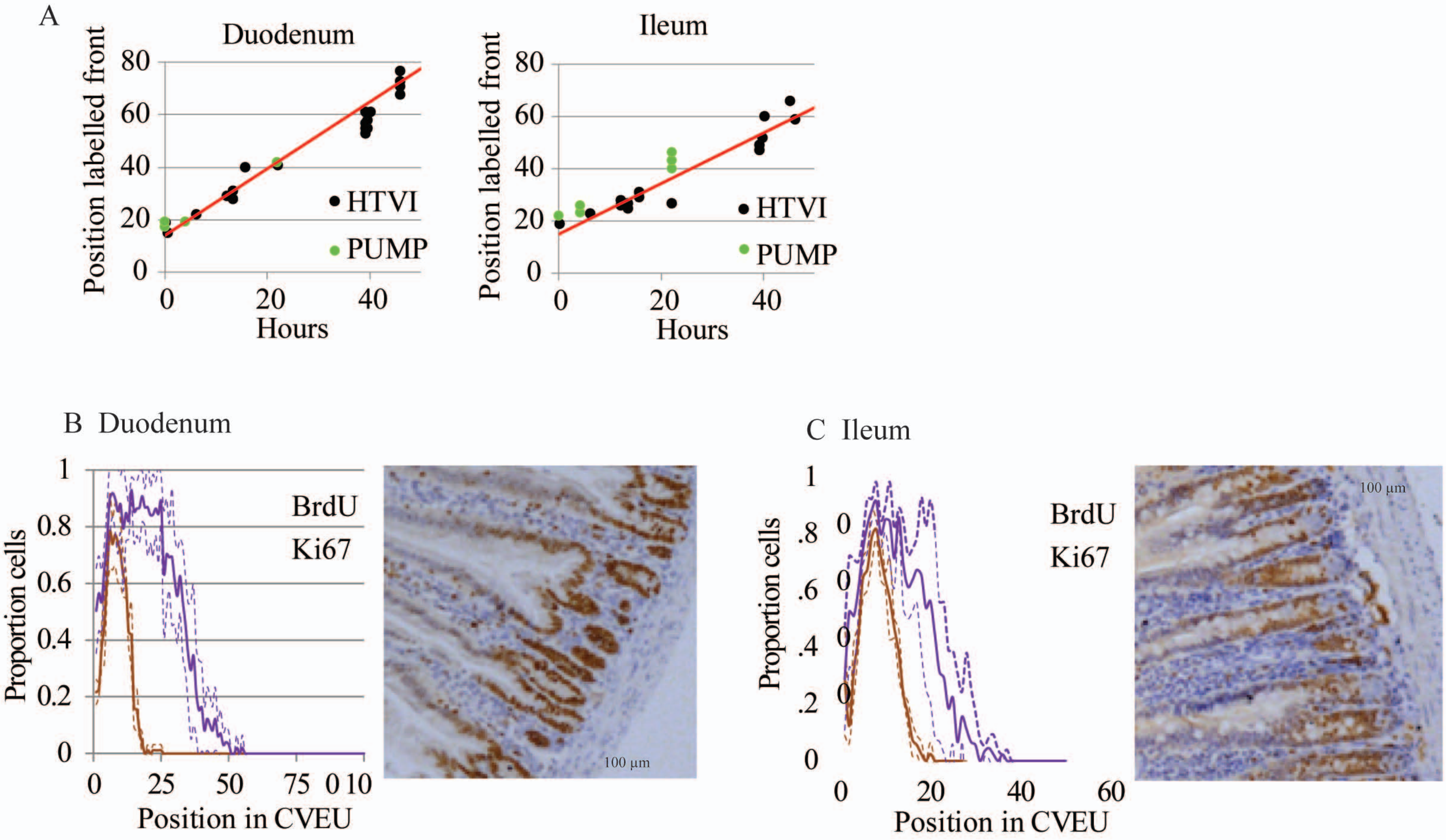

Figure S3 A) Position of the BrdU-labelled front over time on the CVEU in chronic inflammation mouse models mediated by a TNF producing plasmid (black symbols) and osmotic pump delivered TNF (green symbols) in duodenum and ileum. The red line denotes the model in equation [1] fitted to the dataset generated with the TNF plasmid mediated chronic mouse model. B-C) Proportion of Ki-67-positive and BrdU-labelled cells, 2 h after its administration, at each position of the CVEU in duodenum and ileum of healthy mice. Continuous lines show the mean value and discontinuous lines represent the standard error interval estimated from 3-5 animals. Images show Ki-67-positive cells (dark brown) on the villus of duodenum (B) and ileum (C) of healthy mice. Ki-67 protein is reportedly not detected in non-proliferative cells [52,53]. However, the labelling profiles in the duodenum (B) and ileum (C) of healthy mice indicated that we were detecting Ki-67 protein on the villi, at positions where proliferative epithelial cells have not previously been reported. The most plausible explanation for the widespread of Ki-67 labelling is that this protein is present and detectable (not yet degraded) in cells for a period after exiting the division cycle, which is supported by recent findings [54]. We hypothesise that if cell migration is relatively fast, non-proliferative Ki-67 positive cells, which have recently and rapidly ceased proliferation, could be detected on the villus. Supporting this explanation, we found proliferative cells at higher positions of the CVEU in duodenum, where cell proliferation and migration are faster [30,32] than in ileum. These results suggest that Ki-67 detection does not provide an accurate estimation of the size of the proliferative compartment in mouse small intestinal epithelium.

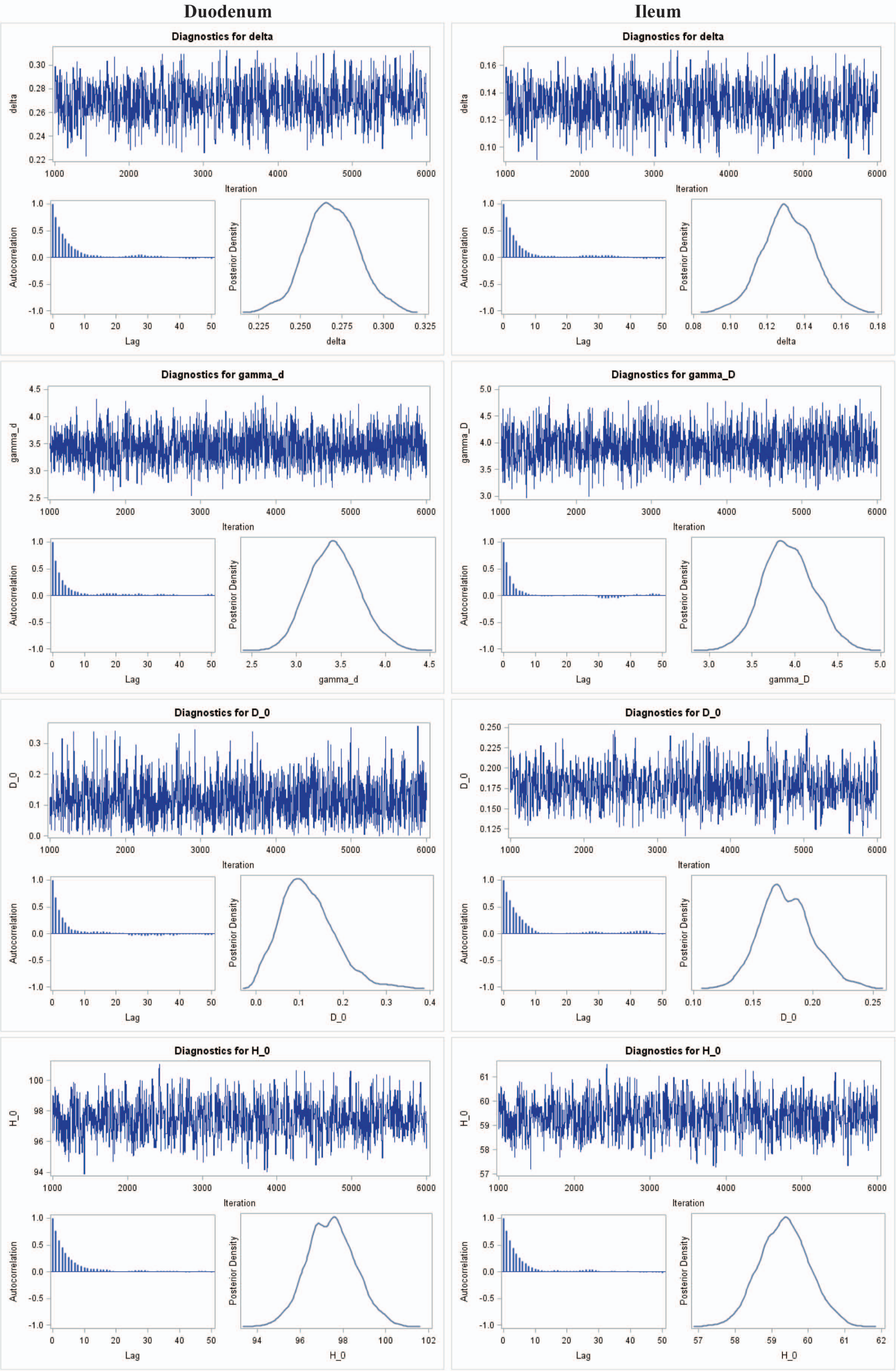

Figure S4. MCMC diagnosis plots for the estimates of the parameters of model in equation (10). The description of the parameters can be found in Table S3. Panels show three plots for each parameter: traces or parameter value estimated in each step of the MCMC (top); autocorrelation functions for the estimates of the parameter (bottom left) and the parameter posterior distribution (bottom right).

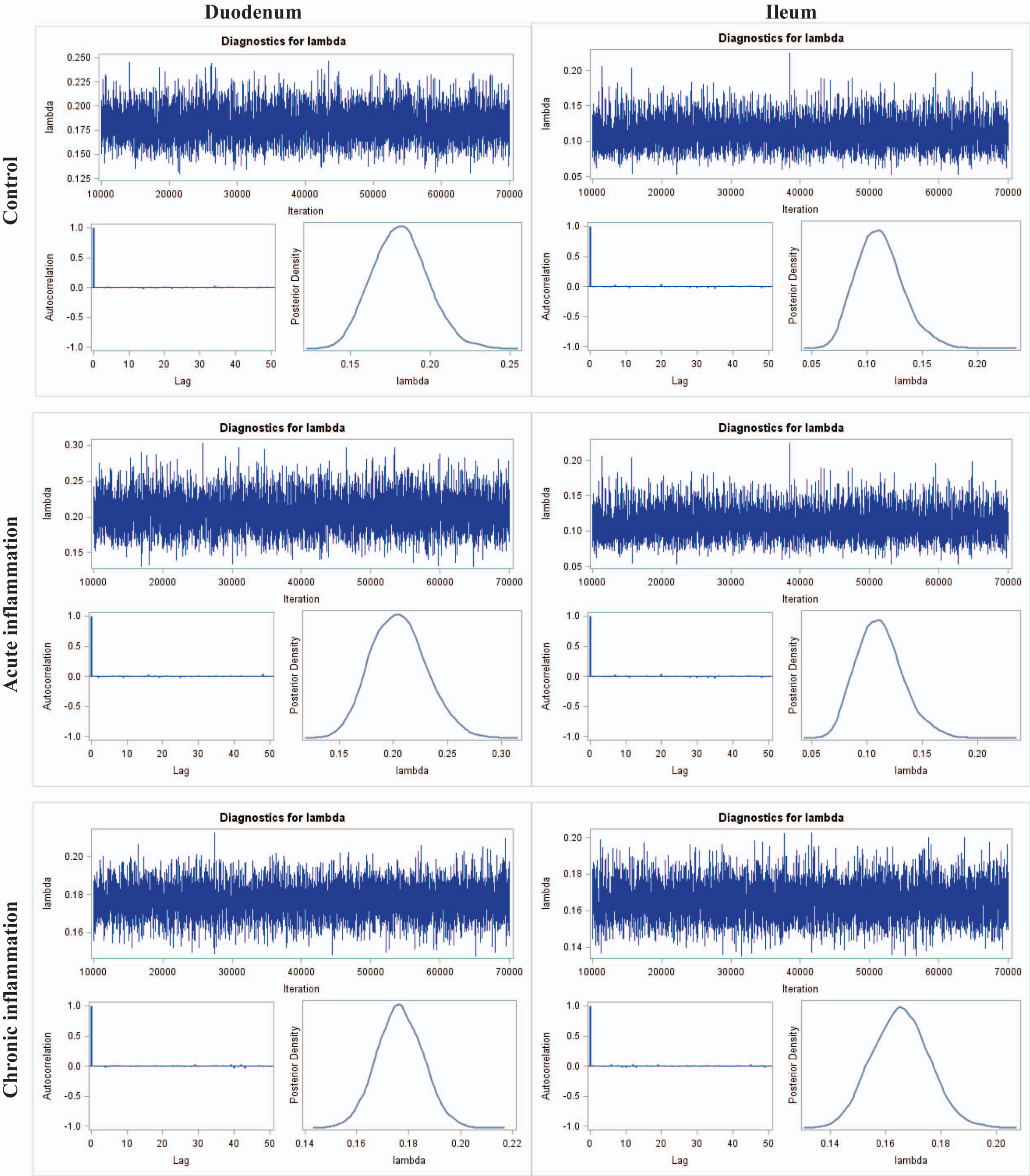

Figure S5. MCMC diagnosis plots for the estimates of the λ parameter of the model in equation (26) in duodenum and ileum of our experimental mouse models. Panels show three plots for each parameter: traces or parameter value estimated in each step of the MCMC (top); autocorrelation functions for the estimates of the parameter (bottom left) and the parameter posterior distribution (bottom right).
